# Extrasynaptic Glutamate Receptor Activation as Cellular Bases for Dynamic Range Compression in Pyramidal Neurons

**DOI:** 10.3389/fphys.2012.00334

**Published:** 2012-08-24

**Authors:** Katerina D. Oikonomou, Shaina M. Short, Matthew T. Rich, Srdjan D. Antic

**Affiliations:** ^1^Department of Neuroscience, University of Connecticut Health CenterFarmington, CT, USA

**Keywords:** prefrontal cortex, pyramidal neuron, synaptic integration, dendritic spike, voltage-sensitive dye

## Abstract

Repetitive synaptic stimulation overcomes the ability of astrocytic processes to clear glutamate from the extracellular space, allowing some dendritic segments to become submerged in a pool of glutamate, for a brief period of time. This dynamic arrangement activates extrasynaptic NMDA receptors located on dendritic shafts. We used voltage-sensitive and calcium-sensitive dyes to probe dendritic function in this glutamate-rich location. An excess of glutamate in the extrasynaptic space was achieved either by repetitive synaptic stimulation or by glutamate iontophoresis onto the dendrites of pyramidal neurons. Two successive activations of synaptic inputs produced a typical NMDA spike, whereas five successive synaptic inputs produced characteristic plateau potentials, reminiscent of cortical UP states. While NMDA spikes were coupled with brief calcium transients highly restricted to the glutamate input site, the dendritic plateau potentials were accompanied by calcium influx along the entire dendritic branch. Once initiated, the glutamate-mediated dendritic plateau potentials could not be interrupted by negative voltage pulses. Activation of extrasynaptic NMDA receptors in cellular compartments void of spines is sufficient to initiate and support plateau potentials. The only requirement for sustained depolarizing events is a surplus of free glutamate near a group of extrasynaptic receptors. Highly non-linear dendritic spikes (plateau potentials) are summed in a highly sublinear fashion at the soma, revealing the cellular bases of signal compression in cortical circuits. Extrasynaptic NMDA receptors provide pyramidal neurons with a function analogous to a dynamic range compression in audio engineering. They limit or reduce the volume of “loud sounds” (i.e., strong glutamatergic inputs) and amplify “quiet sounds” (i.e., glutamatergic inputs that barely cross the dendritic threshold for local spike initiation). Our data also explain why consecutive cortical UP states have uniform amplitudes in a given neuron.

## Introduction

**Figure 1:**
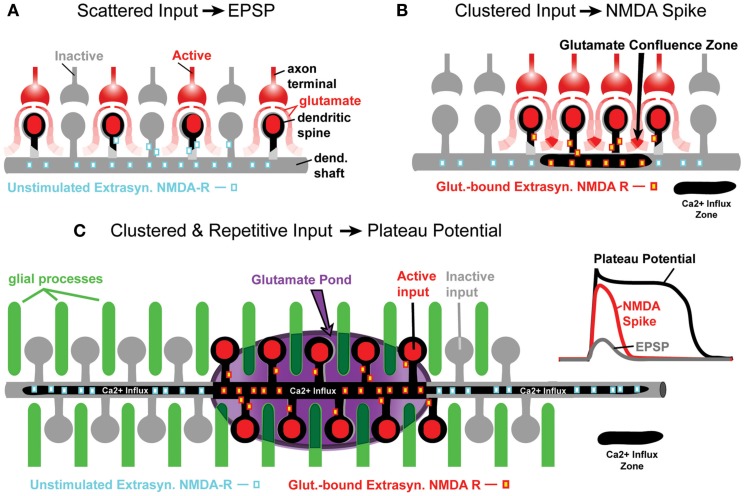
**Glutamate processing in spiny neurons**. **(A)** Sparse glutamatergic inputs fail to activate extrasynaptic NMDA receptors located on dendritic shafts and spine necks. Ca^2+^ influx zone (black contour) restricted to spine heads only. **(B)** Clustered synaptic activity is an effective stimulus for extrasynaptic NMDA receptors, because glutamate spilled from several neighboring synapses summates between active dendritic spines (glutamate confluence zone). Ca^2+^ influx zone now contains the input receiving spines and a short segment of the dendritic shaft (black contour). **(C)** The gray contour represents a segment of a spiny dendrite. Glial processes (green cylinders) are interposed between dendritic spines. Darker green indicates less glutamate uptake in glial process, or release of glutamate from the glial process. For a period of time, an entire segment of the spiny dendrite is surrounded by extracellular matrix rich in glutamate (“glutamate pond”) – marked by purple ellipsoid. The Ca^2+^ influx zone now includes spines and dendritic shaft below spines. The Ca^2+^ influx occurring proximally and distally to the clustered synaptic input site is due to bi-directional propagation of dendritic plateau potential, which initiated inside the glutamate pond. Inset traces: schematic representation of dendritic voltage waveforms associated with EPSPs, NMDA spikes, and plateau potentials.

Each pyramidal neuron in the mammalian cerebral cortex receives several thousands of glutamatergic synapses (Elston, 2003), over 2/3 of which impinge on basal and oblique dendrites (Larkman, 1991). Being directly connected to the cell body, the basal dendrites are in a prime location for delivering large amounts of depolarizing current to the cell body (Oakley et al., 2001; Benucci et al., 2004; Enoki et al., 2004; Milojkovic et al., 2004). In principle, one can identify two parts of the depolarizing current in basal dendrites. One part of this current is of synaptic origin; mediated by the opening of ion channels in dendritic spines (Yasuda et al., 2003; Nimchinsky et al., 2004). The second portion of this current is mediated by the opening of voltage-gated and ligand-gated membrane conductances located on the dendritic shafts of basal dendrites (Schiller et al., 1995; Enoki et al., 2004; Milojkovic et al., 2005b; Kampa and Stuart, 2006; Acker and Antic, 2009; Chalifoux and Carter, 2011). The contributions from these two sources of depolarizing current (synaptic and extrasynaptic) vary with the amount of glutamatergic input. The total glutamatergic input in one dendritic region is determined by the frequency of afferent activity (temporal summation of glutamate) or convergence of afferent axon terminals into the same region (spatial summation of glutamate). When glutamatergic inputs are sparse and scattered (Swadlow and Hicks, 1997), tiny amounts of glutamate that spill out from synaptic clefts are quickly removed from the extracellular space by astrocytic processes (Figure 1A); this mechanism is especially effective at physiological temperatures (Asztely et al., 1997; Kullmann et al., 1999). Clustered synaptic activity (Figure 1B), on the other hand, overcomes the astrocytic ability to remove glutamate, allowing the excitatory neurotransmitter to freely exit loose synaptic clefts (Herman et al., 2011) and reach areas of the neuronal membrane between dendritic spines (Chalifoux and Carter, 2011). To a first approximation, an astrocytic process can handle glutamate from one active spine (Figure 1A), but it fails to completely remove glutamate from the extrasynaptic space when many neighboring spines are active at the same moment of time (Figure 1B). The residual glutamate then becomes available to activate extrasynaptic NMDA receptors (Tovar and Westbrook, 1999). Upon just two consecutive activations of synaptic inputs the conditions are met to trigger a local dendritic spike (Polsky et al., 2004), fully mediated by NMDA receptor channels (Schiller et al., 2000; Major et al., 2008).

Initially, the NMDA spike was thought to be a pure voltage spike (Schiller et al., 2000; Rhodes, 2006), just like the axonal action potential (AP; Hodgkin and Huxley, 1952). This was based on the fact that both Na^+^ channels and NMDA receptor channels exhibit a region of negative slope conductance, which is the biophysical substrate of regenerative property (Mayer et al., 1984; Nowak et al., 1984). We now think that NMDA spikes are not solely based on the summation of voltage (Gasparini et al., 2004; Rhodes, 2006), but rather on the summation of glutamate contributed by many individual boutons that converge in a small space (Figure 1B). In support of this view, it has recently been shown that glutamatergic synapses are functionally clustered on developing dendrites. Synapses that are located near each other on the same dendritic branch exhibit a higher degree of temporal correlation than synaptic pairs on different dendrites (Kleindienst et al., 2011). Spatio-temporal clustering of synaptic inputs (Larkum and Nevian, 2008) and activation of extrasynaptic receptors due to this clustering (Suzuki et al., 2008; Chalifoux and Carter, 2011) are broadening the realm in which we view information processing in the central nervous system (Poirazi et al., 2003; Antic et al., 2007; Magee, 2011; De-Miguel and Fuxe, 2012).

In the present paper, we will compare NMDA spikes with glutamate-evoked plateau potentials (Antic et al., 2010), and argue that both events are mediated by the extrasynaptic NMDA receptors (Schiller et al., 2000; Polsky et al., 2009; Chalifoux and Carter, 2011). In spite of many similarities, these two events show some important differences. Compared to NMDA spikes, the glutamate-mediated dendritic plateau potentials are larger in amplitude and duration. Unlike the NMDA spike, the dendritic plateau potential depolarizes the cell body beyond the AP threshold and lasts for more than 100 ms (Oakley et al., 2001; Wei et al., 2001; Cai et al., 2004; Milojkovic et al., 2004, 2005a; Major et al., 2008). During an intense outburst of cortical activity, glutamate released from multiple axon terminals completely overcomes the ability of astrocytic processes to clear extrasynaptic glutamate (Parpura et al., 1994). Not only does the uptake of glutamate in astrocytic processes begin to significantly slow down, but also, in response to repetitive afferent activity, the astrocytes begin to actively release glutamate back into the extracellular matrix (Parpura et al., 1994; Jourdain et al., 2007; Min and Nevian, 2012). For a brief period of time, the dendritic shaft, and accompanying dendritic spines are submerged in a pool of glutamate (Figure 1C). This temporary arrangement triggers a qualitatively different signal compared to a classical NMDA spike. The glutamate-evoked plateau potential is unique in two physiological aspects: (1) somatic membrane potential and (2) dendritic calcium dynamics. Dendritic plateau potentials initially start as NMDA spikes (Milojkovic et al., 2004, 2005a; Major et al., 2008). Upon conversion from NMDA spike to dendritic plateau potential, the somatic voltage waveform is no longer like a large, pointy EPSP (Polsky et al., 2004); it becomes a more sustained depolarization event, reminiscent of a cortical UP state (Milojkovic et al., 2004, 2005a; Antic et al., 2007). After this transition, the dendritic calcium signal switches from a highly localized calcium transient characteristic of NMDA spikes (Schiller et al., 2000; Holthoff et al., 2004; Chalifoux and Carter, 2011; Katona et al., 2011) to a robust calcium flux that engulfs the entire dendritic branch (Milojkovic et al., 2007; Major et al., 2008; Figure 1C, black contour). During intense cortical activity, it is highly probable that two plateau potentials may coincide in the dendritic tree of a pyramidal neuron. Here, we studied how two dendritic plateau potentials occurring simultaneously in two basal dendrites summate in the cell body.

## Materials and Methods

### Brain slice and electrophysiology

Sprague Dawley rats (P21–42) were anesthetized with isoflurane inhalation, decapitated, and the brains were extracted with the head immersed in ice-cold, artificial cerebrospinal fluid (ACSF), according to an animal protocol approved by the Center for Laboratory Animal Care, University of Connecticut. ACSF contained (in mM) 125 NaCl, 26 NaHCO_3_, 10 glucose, 2.3 KCl, 1.26 KH_2_PO_4_, 2 CaCl_2_, and 1 MgSO_4_, pH 7.4. Coronal slices (300 μm) were cut from frontal lobes. All experimental measurements were performed at 32–34°C. Whole-cell recordings were made from visually identified layer 5 pyramidal neurons within the ventral medial PFC, including prelimbic and infralimbic cortex. Intracellular solution contained (in mM) 135 *K*-gluconate, 2 MgCl_2_, 3 Na_2_-ATP, 10 Na_2_-phosphocreatine, 0.3 Na_2_-GTP, and 10 HEPES (pH 7.3). Electrical signals were amplified with Multiclamp 700B and digitized with two input boards: (1) Digidata Series 1322A (Molecular Devices, Union City, CA, USA) and (2) Neuroplex (RedShirtImaging, Decatur, GA, USA). Only cells with a membrane potential more negative than −50 mV (not corrected for junction potential), and AP amplitudes exceeding 70 mV (measured from the baseline) were included in this study. APs were evoked with depolarizing current steps injected into the cell body (intensity 150–300 pA, duration 250 ms). Sodium channel blocker Tetrodotoxin (TTX) and NMDA receptor blocker dl-2-Amino-5-phosphonopentanoic acid (APV) were purchased from Sigma (St. Louis, MO, USA) and used at final concentrations of 1 and 50 μM, respectively.

### Dye injection

Voltage-sensitive dye (JPW-3028, synthesized by Dr. Leslie Loew, University of Connecticut Health Center), and calcium-sensitive dyes (Ca-Green-1, Oregon Green BAPTA-1, and bis-fura-2; purchased from Invitrogen, Carlsbad, CA, USA) were dissolved in intracellular solution. The protocol for voltage-sensitive dye injection has been previously described (Antic, 2003). Briefly, neurons were filled through whole-cell patching pipettes with JPW-3028 for 45 min. Dye-free solution was at the tip of the pipette, while the back of the pipette lumen was loaded with dye-rich solution (400–800 μM). After 45 min of loading with voltage-sensitive dye, the filling pipette was pulled out (outside-out patch) and brain slices were left to incubate for 1–3 h at room temperature, allowing the dye to diffuse into dendritic tree. Right before optical recordings the cells were re-patched with a dye-free pipette. In calcium imaging experiments, Alexa Fluor 594 (50 μM) was included in the intracellular solution to aid the visually guided positioning of glutamate electrodes onto dendrites. The calcium-sensitive dyes (100–200 μM) were injected for 30–45 min before optical recordings.

### Dendritic voltage and calcium imaging

Multi-site dendritic imaging was performed on either Olympus BX51WI or Zeiss, Axioskop FS microscope, equipped with UV-compatible 40× objective, two camera ports, and a low-ripple Xenon arc lamp (OptiQuip, Highland Mills, NY, USA) for epi-illumination. Functional dendritic imaging was performed with a NeuroCCD camera (80 × 80 pixels, RedShirtImaging, LLC, Decatur, GA, USA). Voltage-sensitive dye signals were sampled at 1,000 and 500 Hz frame rates. At these sampling rates the AP voltage waveforms riding on top of dendritic plateau potentials were severely under sampled. However, in this project we were focused on the slow component of dendritic depolarizations, which was accurately represented at these sampling rates (Figure 3B, ROI). Calcium-dye signals were sampled at 500 Hz full frame rate. Neutral density filters were used to reduce the intensity of epi-illumination light during positioning and focusing. Duration of illumination epochs were kept to minimum: Ca^2+^ imaging 2,500–5,000 ms; voltage imaging 500–2,500 ms. In spite of all precautions, after ~16 optical recording sweeps, on average, neurons began to show an increase in AP half-width due to phototoxicity (Antic et al., 1999). At that point experiments were stopped. Optical filters were purchased from Chroma Technology (Rockingham, VT, USA) and Omega Optical (Brattleboro, VT, USA). Filters for Alexa Fluor 594 were a Chroma exciter HQ580/20× (570–590 nm bandpass), dichroic Q595LP, and emitter HQ630/60 m (600–660 nm bandpass). The filter cube for Ca-Green-1 and Oregon Green BAPTA-1 contained an Omega exciter 500AF25, dichroic 525DRLP, and emitter 530ALP. The filter set for voltage imaging (JPW-3028) consisted of a Chroma exciter D510/60 (480–540 nm bandpass), dichroic 570dcxru, and emitter E600lp (600 nm longpass). The filter cube for bis-fura-2 contained a Chroma exciter D380/30× (365–395 nm bandpass), dichroic 400dclp, and emitter E470lp (470 nm longpass).

### Stimulations

Stimulation electrodes were pulled from borosilicate glass with filament (1.5 mm outer diameter). Synaptic stimulation pipettes (7 MΩ) were filled with regular ACSF and glutamate stimulation pipettes (40–50 MΩ) were filled with 200 mM Na-glutamate (pH = 9). A programmable stimulator, Master-8, and a stimulus isolation unit, IsoFlex (A.M.P.I., Jerusalem, Israel), were used to generate current pulses for both synaptic stimulation and glutamate iontophoresis. Following the spread of fluorescent dyes into the dendrites, the stimulation pipettes were positioned in mid-regions of basal branches; between 65 and 130 μm from the cell body. Navigation of the flexible glass pipettes through the slice tissue was achieved with the aid of a “fourth axis” (concomitant engagement of both *X* and *Z* axis), available on Sutter Instruments M-285 motorized micromanipulator. The intensities of current pulses for synaptic stimulation (two to five pulses, 50 Hz, 100 μs, 50–90 μA), or of single glutamate microiontophoresis puffs (5 ms, 0.9–2.5 μA), were adjusted to produce a long-lasting somatic depolarization (half-width 200–500 ms) crowned by two to six APs (UP-state-like depolarization). The reported stimulation current intensities are nominal values obtained from the IsoFlex settings.

### Data analysis

**Figure 2:**
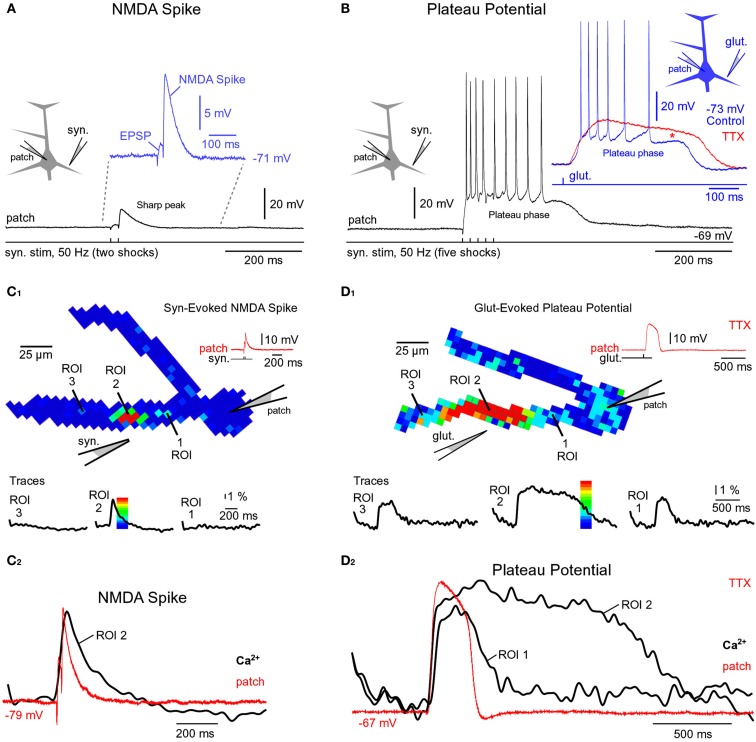
**Comparisons of NMDA spike and plateau potential**. **(A)** Schematic drawing of the experimental outline. “Patch” marks somatic whole-cell recordings. Two synaptic shocks produce an EPSP followed by the NMDA spike. Inset: blowup of the same event. In this and all of the following figures the resting membrane potential is displayed next to the trace. **(B)** Five synaptic shocks (50 Hz) produce a dendritic plateau potential ~300 ms in duration and ~20 mV in amplitude. Same voltage and time scales in **(A,B)**, black traces. Inset: glutamate iontophoresis (duration 5 ms) in mid segment of a basal dendrite produces a glutamate-evoked plateau potential (blue trace). Block of sodium channels by bath application of TTX revealed the underlying slow component (red trace). Red asterisk marks a TTX-induced increase in plateau amplitude. **(C_1_)** Multi-site optical imaging reveals spatial distribution of calcium transients in two basal dendrites at the peak of the synaptically evoked NMDA spike. Ca^2+^ signal amplitude is color-coded according to the scale shown superimposed on the trace. Traces: three ROIs (5–8 pixels spatially averaged) were selected for display. Note that Ca^2+^ transient is absent from dendritic segments proximal (ROI 1) and distal (ROI 3) to the synaptic input site (ROI 2). **(C_2_)** Somatic whole-cell recording (red) and dendritic calcium signal (black) are copied from **(C_1_)** and superimposed. **(D_1_)** Same as in **(C_1_)**, except glutamate iontophoresis (5 ms) was used to trigger dendritic plateau potential (duration ~300 ms). Robust Ca^2+^ transients are present proximally and distally to the glutamate input site (ROI 2). Note that Ca^2+^ transients in ROI 1 and ROI 3 have different temporal dynamics than the transient obtained at the glutamate input site (ROI 2). **(D_2_)** Same as in **(C_2_)** except signals copied from **(D_1_)**.

**Figure 3:**
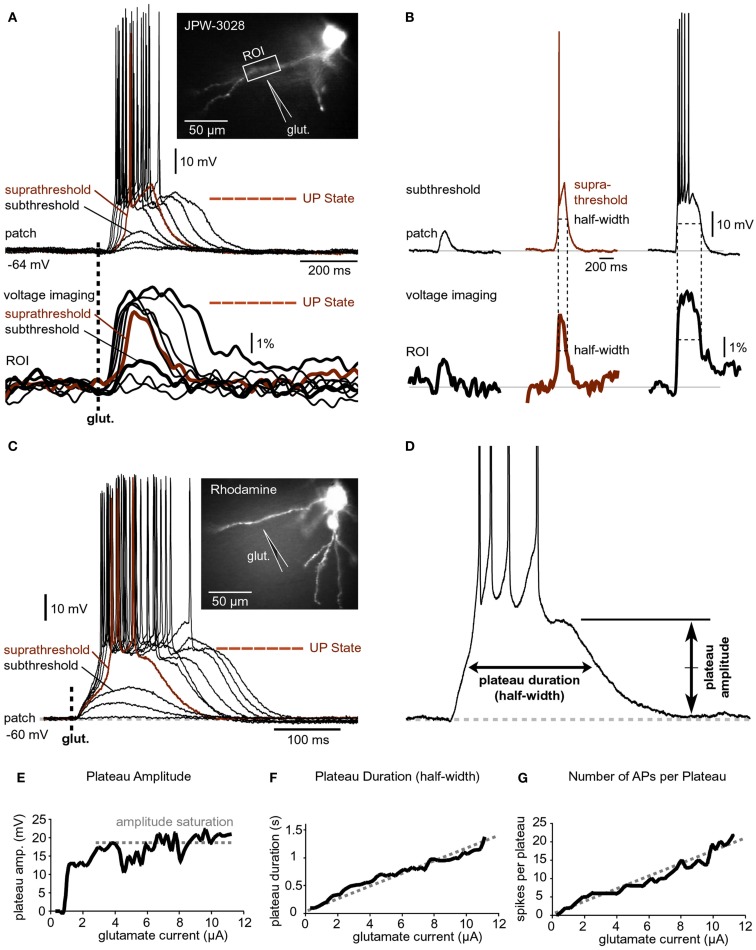
**Input-output functions**. **(A)** A pyramidal neuron was injected with the voltage-sensitive dye (JPW-3028) and stimulated by glutamate microiontophoresis (inset). Intensity of glutamate iontophoretic current was gradually increased (fixed increment), while dendritic depolarizations were recorded optically in dendrite (ROI) and electrically in the soma (patch). Brown color marks a sudden jump – spike. **(B)** Three experimental sweeps selected from **(A)** are aligned individually for comparisons between signal half-widths in dendrite (ROI) and in soma (patch). **(C)** Same as in **(A)** except rhodamine was injected to trace dendrites. **(D)** Plateau amplitude and plateau duration were measured from the slow component of the somatic electrical signal. Different cell than in **(C)**. **(E–G)** Plateau amplitude, plateau duration, and number of APs per plateau are plotted against the intensity of glutamate stimulus. Different cell than in **(D)**.

Analysis of optical data, including spatial averaging, high pass, and low pass filtering, was conducted with Neuroplex 8.0.0 (RedShirtImaging). To process off-line calcium imaging data, we applied a Butterworth high pass filter at 0.1 Hz cut-off and a Gaussian low pass filter at 30 Hz cut-off; for voltage imaging data, the high and low pass filters were set at Butterworth 0.1 Hz and Gaussian 150 Hz, unless otherwise specified. Electrical recordings were analyzed in Clampfit 9 (Molecular Devices, Sunnyvale, CA, USA). Plateau amplitude, also termed “amplitude of the slow component of the somatic depolarization,” was measured from base line (resting membrane potential) to the shoulder following AP burst (Figure 3D, plateau amplitude). If the depolarization “shoulder” was masked by APs, plateau amplitude was measured from base line to the trough between the last two APs in the burst. Plateau amplitude in TTX-treated neurons was measured in the last 1/4 of the plateau phase, to avoid the broad peaks that were often prominent in the first half of the plateau phase (Figure 2B, Inset, red trace). Plateau duration was measured at half amplitude of the slow component of somatic depolarization (Figure 3D, plateau amplitude). The success rate of plateau initiation was systematically studied in five pyramidal neurons by delivering synaptic shocks on multiple basal dendrites. Synaptically evoked plateau depolarizations with amplitude less than 8 mV, or duration less than 100 ms, were considered failures. In experiments with TTX, the neurons were stimulated with three glutamate pulses (1 Hz). In the paired *t*-test analysis of the TTX data the first glutamate pulse in control condition (before TTX) was compared with the first glutamate pulse in the test condition (after TTX). The same was done for the second and third glutamate pulses, respectively. Experimental data on the summation of subthreshold plateau potentials (Figure 10) was divided in three groups. In the first group termed “*Two Small Resulting in no AP*” (Figure 10A), each summand in the summation experiment was less than 10.2 mV before summation (the full range of peak amplitudes in this group was from 4.2 to 10.2 mV). The critical criteria for sorting data into this group were that both summands in each pair of summands were in the lower part of the amplitude range (less than 10.2 mV), and that somatic APs were not generated upon summation. This strategy resulted in the formation of a group in which the average arithmetic sum of summand pairs was only 14.8 ± 0.74 mV (*n* = 13 experiments). The entire group, consisting of all plateau amplitudes before summation, is plotted in Figure 10D, *Small no AP*. In the second group termed “*Two Large Resulting in no AP*,” all individual summands had a peak amplitude greater than 8.4 mV before summation, and somatic APs were not generated (Figure 10B). This strategy resulted in the formation of the second data group in which the average arithmetic sum of summand pairs was 21.7 ± 0.67 mV (*n* = 16 experiments). The full range of peak amplitudes in the second group was from 8.4 to 18.2 mV (Figure 10D, *Large no AP*). In the third group termed “*Two Large Resulting in 1 or 2 AP*,” all individual summands had a peak amplitude greater than 7 mV (range 7.1–18.8 mV). The average arithmetic sum of summand pairs in the third group was 22.6 ± 0.96 mV (*n* = 25 experiments). The critical criterion for sorting data into the third group was that one or two APs accompanied the physiological sum (Figure 10C). The entire group 3, including all plateau amplitudes before summation, is plotted in Figure 10D, *Large Resulting in APs*. Statistical tests were performed using SigmaPlot 8.02 (Systat Software, San Jose, CA, USA). All statistics were done on raw data points before normalization unless otherwise specified. Paired Student’s *t*-tests were used for comparing data obtained from the same neuron (in two different conditions). Unpaired Student’s *t*-test was used for data obtained from different neurons. Significance was set at *p* < 0.05 (one asterisk), and high significance at *p* < 0.01 (two asterisks). Values are presented as mean ± SEM.

## Results

### Comparisons between nmda spikes and plateau potentials

We studied glutamate-mediated dendritic spikes in layer 5 pyramidal neurons using a combination of whole-cell recordings, synaptic stimulation, glutamate microiontophoresis, calcium imaging, and voltage-sensitive dye imaging. Dendritic NMDA spikes were triggered by two synaptic shocks (50 Hz) delivered in the vicinity of basal dendrites (Figure 2A, Drawing). Synaptically evoked dendritic NMDA spikes had an average amplitude of 9.3 ± 0.4 mV (*n* = 12 neurons, *N* = 27 trials), when measured with patch electrodes in the soma (Figure 2A, patch). Increasing the number of synaptic shocks from two to five caused a dramatic qualitative change in the voltage waveform. A train of five synaptic shocks (50 Hz) triggered plateau potentials, characterized with a rapid onset, somatic amplitude of (15.7 ± 0.9 mV), and plateau phase which lasted 260 ± 27 ms (*n* = 35 trials in 15 neurons), followed by a rapid collapse back to the resting membrane potential (Figure 2B). Success rates of NMDA spike and plateau potential initiations were systematically studied in five pyramidal neurons by delivering synaptic shocks on multiple basal dendrites. While the NMDA spike initiation was successful in ~100% of dendrites tested (19 out of 20 dendrites), the success rate of synaptically evoked plateau potentials was notably lower, ~37% (7 out of 19 dendrites). Nevertheless, the successful recordings of plateau potentials (Figure 2B) show that synaptic terminals activated by a single electrode can release a sufficient amount of glutamate to depolarize pyramidal neurons for hundreds of milliseconds and generate bursts of AP firing. An equivalent depolarization plateau, characterized by 15–20 mV amplitude (slow component) and ~300 ms duration was triggered by a single 5-ms iontophoretic pulse of glutamate delivered in the middle segment of a basal dendrite (65–130 μm from the soma) in all neurons tested in this way (*n* = 20, success rate = ~100%; Figure 2B, Inset, blue trace).

Besides differences in voltage waveforms (Figures 2A,B, black traces), dendritic NMDA spikes, and dendritic plateau potentials may also differ in respect to the dendritic calcium transients that accompany them. To test this hypothesis, in the next series of experiments pyramidal neurons were injected with calcium-sensitive dyes and calcium signals were sampled simultaneously from the entire length of a basal branch. Multi-site recordings provide a more complete account of the spatial aspect of dendritic physiology than laser scanning methods, which explore one dendritic spine at a time (Yasuda et al., 2003), or one spine and one spot on the dendritic shaft in the immediate vicinity of that spine (Chalifoux and Carter, 2011). Wide-field calcium imaging revealed that during a synaptically evoked NMDA spike, dendritic segments proximal to the synaptic input site (Figure 2C_1_, ROI 1), or distal to the input site (Figure 2C_1_, ROI 3), did not experience any detectable changes in the internal calcium ion concentration (*n* = 11 out of 11 basal dendrites tested). The dendritic calcium influx was restricted to a very short dendritic segment, directly opposed to the synaptic stimulation electrode (Figure 2C_1_, ROI 2). In these cells, the length of the dendritic segment experiencing an NMDA spike-associated calcium influx was on average 22.7 ± 2 μm (*n* = 11 basal dendrites in 11 neurons), consistent with glutamate-evoked NMDA spikes (Schiller et al., 2000; their Figure 1D). Durations of dendritic calcium transients and corresponding somatic depolarizations were very similar (Figure 2C_2_). The ratio between dendritic calcium signal half-width and somatic voltage signal half-width was 1.38 ± 0.23 (*n* = 127 sweeps obtained in 11 cells), consistent with two-photon imaging data (Chalifoux and Carter, 2011).

The same wide-field optical imaging method used for NMDA spikes (Figure 2C) was next used to characterize dendritic calcium influx during glutamate-evoked plateau potentials in basal dendrites. Brain slices were bathed in 1 μM TTX to remove the calcium influx due to backpropagating APs. Plateau potentials were triggered by iontophoretically ejecting glutamate (duration = 5 ms) in the middle segment of a basal dendrite. The temporal and spatial profiles of dendritic calcium transients during plateau potentials (Figure 2D) were very different from those observed during NMDA spikes (Figure 2C). The half-widths of dendritic calcium signals at the glutamate input site (Figure 2D_2_, ROI 2, black trace) were on average 5.19 ± 0.4-fold (*n* = 8) greater than the half-widths of somatic voltage signals recorded by the patch pipette (red trace). In all neurons tested in this way (eight out of eight), dendritic segments proximal (Figure 2D_1_, ROI 1), and distal (Figure 2D_1_, ROI 3) to the glutamate input site experienced robust calcium transients. The durations of dendritic calcium transients outside the glutamate input site were similar to the duration of the voltage waveform obtained in the cell body (Figure 2D_2_, compare ROI 1 and patch). More specifically, the ratio of the dendritic calcium signal half-width to the somatic voltage signal half-width was 1.63 ± 0.12 (*n* = 8) for dendritic segments 60 μm more distal to the glutamate input site and 1.37 ± 0.10 (*n* = 8) for dendritic segments 60 μm more proximal to the glutamate input site. These results show that dendritic NMDA spikes and dendritic plateau potentials can be distinguished based on three principle differences: (1) voltage waveforms in the cell body; (2) calcium dynamics at the glutamate input site; and (3) spatial distribution of calcium transients along the input receiving branch.

### Dendritic up states drive somatic depolarized states

A step-wise increase in the amount of iontophoretically applied glutamate was used to model glutamatergic inputs that may be received by basal dendrites of PFC pyramidal neurons at different levels of afferent presynaptic activity (Figure 3A). Glutamate iontophoretic current was increased gradually in fixed increments (0.1 or 0.2 μA), while glutamate-evoked potentials were recorded simultaneously in cell body (patch) and in dendrites using voltage-sensitive dye (Figure 3A, ROI). A transition from subthreshold to suprathreshold dendritic potential (dendritic spike) was invariably accompanied by somatic transition from subthreshold EPSP-like depolarization to plateau potential (Figure 3A, brown trace). A further increase in glutamate stimulation intensity yielded very small increases in dendritic potential amplitude (Figure 3A, voltage imaging), resembling an all-or-none mechanism (spike). Unlike axonal APs, which have precisely uniform durations (half-widths) determined by activation and inactivation kinetics of Na^+^ and K^+^ currents (Hodgkin and Huxley, 1952), the durations (half-widths) of glutamate-evoked dendritic plateau potentials are not fixed by intrinsic membrane properties. The durations of dendritic plateau potentials are variable and increase as the intensity of the glutamatergic input increases (Milojkovic et al., 2005a; Major et al., 2008). When basal dendrites transitioned from resting to a depolarized state (Figure 3A, ROI, UP State), the cell bodies invariably responded to this transition by entering the sustained depolarized state (Figure 3A, patch, UP State). Simultaneous dendritic (voltage-sensitive dye) and somatic (whole-cell) recordings revealed that durations of dendritic plateau potentials (half-widths) were in strict correlation with durations of the corresponding somatic depolarizations (Figure 3B). The ratio of dendritic/somatic duration was 1.009 ± 0.006 (*n* = 184 sweeps in 33 neurons, correlation coefficient = 0.9876). In this data set we included 4 types of experiments: (1) glutamate-evoked plateau potentials with bursts of APs (Figure 3A) *n* = 79 sweeps obtained in 18 pyramidal neurons; (2) glutamate-evoked plateau potentials without APs (*n* = 28 sweeps in 7 cells); (3) synaptically evoked plateau potentials (Figure 2B), *n* = 15 sweeps in nine cells; and (4) glutamate-evoked plateau potentials in TTX (Figure 2B, Inset, red trace), *n* = 62 sweeps in 11 cells. In each of these four groups, the correlation coefficient between dendritic and somatic voltage waveforms (half-width) was greater than 0.97. These data indicate that sustained depolarizations, observed in the cell body during glutamatergic or repetitive synaptic stimulation of basal dendrites, are nothing more than passive reflections of dendritic plateau potentials. What can be potentially interpreted as a neuronal UP state (Figure 3A, patch, UP State) is actually a dendritic UP state (Figure 3A, ROI, UP State) observed from the somatic recording site (Milojkovic et al., 2005a).

Voltage-sensitive dyes are toxic and can influence the membrane currents of cortical pyramidal neurons (Antic et al., 1999). In order to determine whether the toxic effect of voltage-sensitive dyes caused or contributed to the physiological responses described in Figures 3A,B, neurons were injected with the fluorescent marker rhodamine to guide the positioning of glutamate electrodes onto basal dendrites (Figure 3C, image). Glutamate iontophoretic current was increased gradually in fixed increments (0.1 or 0.2 μA), while glutamate-evoked potentials were recorded in the cell body (Figure 3C, patch). Similar input-output functions, including the transitions from subthreshold to suprathreshold depolarizations, were obtained in all rhodamine-filled neurons (five out of five). Plateau amplitude was quantified by measuring the depolarization amplitude after the last AP in the burst, while the plateau duration was measured at half amplitude (Figure 3D). The plot of somatic plateau amplitude versus intensity of dendritic glutamate stimulus showed that plateau amplitudes saturated at low glutamate intensities (Figure 3E). Although the plateau amplitudes were fairly constant throughout a wide range of glutamate intensities (Figure 3E, amplitude saturation), the plateau durations grew linearly with intensity of the dendritic glutamate stimulus (Figure 3F). A linear relationship was also detected between the intensity of glutamate stimulus and the number of APs that accompanied each plateau depolarization (Figure 3G). These data indicate that basal dendrites of PFC pyramidal neurons employ two strategies for coding the intensity of glutamatergic input received in a unit of time: (1) by the amplitude of the slow component of depolarization (Figure 3E); and (2) by duration of the slow component of depolarization (Figure 3F).

### Ionic basis of the dendritic plateau potential

**Figure 4:**
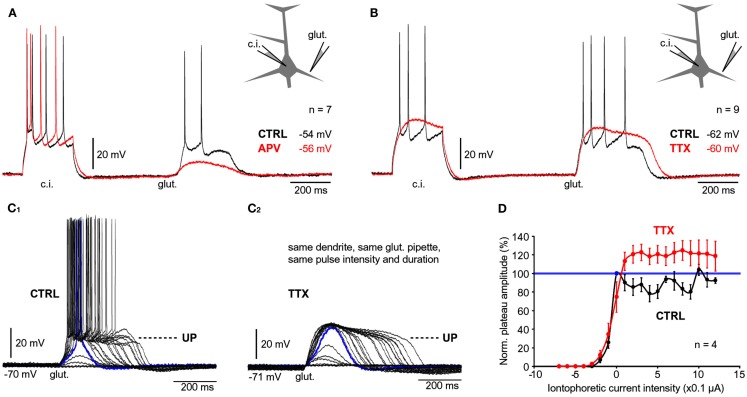
**Contribution of NMDA receptors and sodium channels**. **(A)** Whole-cell recordings of APs evoked by current injection (c.i.) followed by a plateau potential evoked by glutamate iontophoresis (glut.). Recordings were made before (CTRL, black trace) and after application of NMDA receptor antagonist, APV (50 μM, red trace). **(B)** Same as in **(A)** except TTX was used to block VGSC. **(C_1_)** Glutamate electrode was positioned in the middle segment of one basal branch. Intensity of glutamate iontophoretic current was gradually increased (fixed increments), while somatic depolarizations were recorded via whole-cell electrode. **(C_2_)** Same measurements in **(C_1_)**, were repeated in the presence of TTX. **(D)** Amplitude of depolarization is plotted against the intensity of glutamate stimulus before (CTRL, black) and after introduction of TTX (red). All values are normalized against the amplitude of the first plateau in the sequence (blue data point), corresponding to the blue trace in **(C_1_)**.

Application of the NMDA receptor blocker APV (50 μM) resulted in the loss of amplitude and shape of the glutamate-evoked plateau potential (Figure 4A, compare black and red trace). In the presence of APV the amplitude of the slow somatic depolarization was reduced to 57.5 ± 6% (*n* = 7) compared to control measurements obtained in the same neuron (Figure 4A, black trace). A paired *t*-test performed on raw data prior to normalization, determined a statistically significant difference in depolarization amplitudes obtained before and after application of NMDA receptor antagonists (*p* < 0.01, *n* = 7). The excitability of the cell body was not affected by application of APV, as determined by somatic current injections performed during each experimental trial (Figure 4A, c.i.). These data indicate that glutamate-evoked plateau potentials are mediated by NMDA receptor channels.

Sodium channels in basal dendrites of cortical pyramidal neurons support the generation of fast dendritic spikes (Ariav et al., 2003; Milojkovic et al., 2005b). To determine the contribution of Na^+^ channels to the voltage waveforms of glutamate-evoked plateau potentials we stimulated basal dendrites with glutamate pulses (5 ms) before (Figure 4B, black trace) and after bath application of the Na^+^ channel antagonist TTX (1 μM; Figure 4B, red trace). TTX application caused an increase in plateau amplitude (mean amplitude ratio TTX/CTRL = 1.1 ± 0.03, *n* = 27 plateaus from nine cells). A paired *t*-test performed on raw data before normalization determined a statistically significant difference between plateau amplitudes measured in the same neuron before and after application of TTX (*p* < 0.01, *n* = 27). TTX application caused on average a ~21% increase in plateau half-width (average half-width ratio TTX/CTRL = 1.21 ± 0.04, *n* = 27 plateaus from nine cells). Paired *t*-test performed on raw data before normalization determined a statistically significant difference between plateau durations measured in the same neuron before and after application of TTX (*p* < 0.001, *n* = 27). The paradoxical increase in amplitude and duration (Figure 4B, compare black and red trace at the time point marked by “glut.”) can be attributed to the loss of AP-induced K^+^ currents (Bekkers, 2000; Korngreen and Sakmann, 2000; Schaefer et al., 2007).

In the next series of experiments we tested whether the block of Na^+^ current affects the dendritic input-output function. Glutamate iontophoretic current was increased gradually in fixed increments (0.1 or 0.2 μA), while glutamate-evoked potentials were recorded in the cell body before (Figure 4C_1_) and after application of TTX (1 μM; Figure 4C_2_). The amplitudes of the slow component were normalized against the first suprathreshold amplitude in the control sequence (Figure 4C_1_, blue trace, corresponding to 100%) and plotted versus the intensity of the glutamate stimulus (Figure 4D). For each dendrite tested before and after TTX, the transition from subthreshold to suprathreshold dendritic potential (“dendritic spike”) occurred at the same glutamate intensity in both conditions (*n* = 4 dendrites in four neurons), marked by “0 μA” on the graph (Figure 4D). The “control” and “TTX” dendritic input-output functions both exhibited a sigmoidal shape characterized by a clear saturation of the amplitude (Figure 4D), except the peak amplitudes in TTX (red) were consistently greater than in control (black). In both conditions (control and TTX), there was a certain stimulus intensity at which generation of dendritic spike occurred (Figures 4C_1,C_2__, blue traces). A further increase in glutamate stimulation intensity above the intensity required for generation of dendritic spike yielded very small increases in the amplitude of the somatic slow component (Figures 4C_1,C_2__). In the absence of Na^+^ currents, the durations of somatic plateau potentials increased as the intensity of the glutamatergic input increased (Figure 4C_2_). These data indicate that voltage-gated Na^+^ current is not necessary for the transition of dendritic and somatic potentials from Down to UP states.

### Dendritic function in the abundance of glutamate

**Figure 5:**
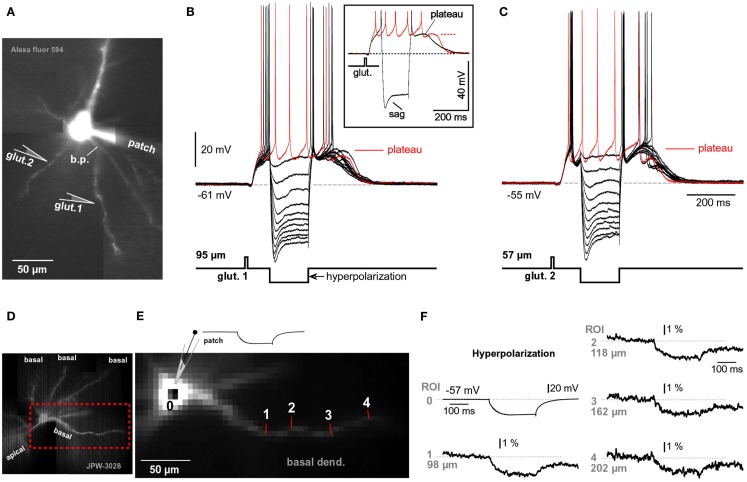
**Hyperpolarization cannot interrupt glutamate-evoked plateau potentials**. **(A)** Pyramidal neuron filled with Alexa Fluor 594. b.p., Branch point. **(B)** A 5-ms glutamate pulse, applied 95 μm from the center of the cell body (glut. 1), triggered a glutamate-evoked plateau potential (red sweep). Next, the glutamatergic stimulation (*glut. 1*) was paired with negative current injections into the soma (black sweeps). Inset: maximal negative current sweep (black) is superimposed with glutamate alone sweep (red). AP, truncated. Characteristic sag is caused by “*h*” current. Gray dashed line – resting potential. Red dashed line – UP state (plateau). **(C)** Same experimental paradigm as in **(B)**, except glutamate application micropipette was positioned closer to the cell body, at location marked by “*glut. 2*,” 57 μm from the soma. **(D)** Microphotograph of a pyramidal cell filled with voltage-sensitive dye JPW-3028. **(E)** Fast camera capture of the region marked by rectangle in **(D)**. Six to nine individual pixels were used for spatial averaging in each region of interest (ROI 1–4). **(F)** Patch clamp recording (ROI 0) is aligned with simultaneous voltage-sensitive dye recordings from four ROIs during somatic hyperpolarization (−36 mV peak amplitude measured in the soma). Each optical trace is a temporal average of four trials.

It has been postulated that local regenerative membrane potentials underlie both phenomena; dendritic NMDA spikes (Schiller et al., 2000; Rhodes, 2006) and dendritic plateau potentials (Milojkovic et al., 2004). The binding of two glutamate molecules converts the ligand-gated NMDA receptor channels into functional voltage-gated channels (Mayer et al., 1984; Nowak et al., 1984). Hyperpolarization, if employed for a sufficient period of time (hundreds of milliseconds) may affect the onset, amplitude, or duration of the NMDA current. Any alterations in dendritic plateau potential would be reflected in somatic voltage waveforms, as determined by voltage imaging (Figures 3A,B). To test if negative voltages can interrupt or in some other way affect plateau potentials, a series of hyperpolarizing current pulses of variable intensities were injected in the middle of a glutamate-evoked plateau potential. Pulse durations were set at >100 ms to allow time for proper charging of the neuronal membrane. The somatic amplitude of hyperpolarization was set to exceed −100 mV. In four out of four neurons tested in this way (Figure 5A), the plateau potentials were not affected by strong hyperpolarization pulses that reached ~50 mV more negative than the resting potential (Figure 5B). After cessation of large negative pulses the somatic membrane potentials swiftly returned to the plateau level, as if hyperpolarizing pulses never occurred (Figure 5B, Inset, black trace). Glutamate-evoked plateau potentials were completely insensitive to hyperpolarizing pulses even when glutamate pipettes were re-positioned to dendritic locations closer to the soma, the source of hyperpolarizing current (Figure 5C). We also applied hyperpolarizing pulses in different phases of the plateau potential (not shown) and found that neither amplitude nor duration of glutamate-evoked dendritic potential could be affected by somatic hyperpolarization to −100 mV (*n* = 4), consistent with Major et al. (2008).

To investigate if hyperpolarizing pulses injected in the soma affect the membrane potential in basal dendrites, we filled pyramidal neurons with voltage-sensitive dyes (Figure 5D) and recorded hyperpolarizing dendritic potentials at multiple sites along basal branches (Figure 5E). Despite low sensitivity of voltage-sensitive dye recordings (~4% for 100 mV change), we were able to detect hyperpolarizing voltage pulses (−25 to −35 mV amplitude at soma) in dendritic segments 140–200 μm away from the soma (Figure 5F, 202 μm), in four out of four pyramidal neurons tested in this way. These data suggest that somatic injections of hyperpolarizing current affect the membrane potential in basal dendrites. The failure of hyperpolarizing somatic pulses to change the amplitude or the duration of dendritic plateau potentials is consistent with the model presented in Figure 1C. Dendritic plateau potentials occur only if dendritic shafts and associated extrasynaptic NMDA receptors are surrounded by a surplus of glutamate ions (Figure 1C, glutamate pond). During an intense glutamatergic drive, with both AMPA and NMDA receptors saturated in synaptic and extrasynaptic surfaces, the input receiving dendritic segment is clamped to the glutamate reversal potential (~0 mV). Upon cessation of the hyperpolarizing pulse the dendritic membrane potential promptly returns to 0 mV, which restores the supply of depolarizing current to the soma (Figure 5B, Inset, black trace). These data also suggest that chemical summation of glutamate in a confined space (Figure 1C) may not only be responsible for the initiation (Major et al., 2008), but also for the maintenance of the plateau phase during glutamate-mediated plateau potentials. That is, the neuron remains in a sustained depolarized state (UP state) as long as one of its basal dendrites is surrounded by a group of glial processes that have stopped absorbing glutamate (Figure 1C, dark green glia).

### Plateau depolarizations in neuronal compartments without spines

**Figure 6:**
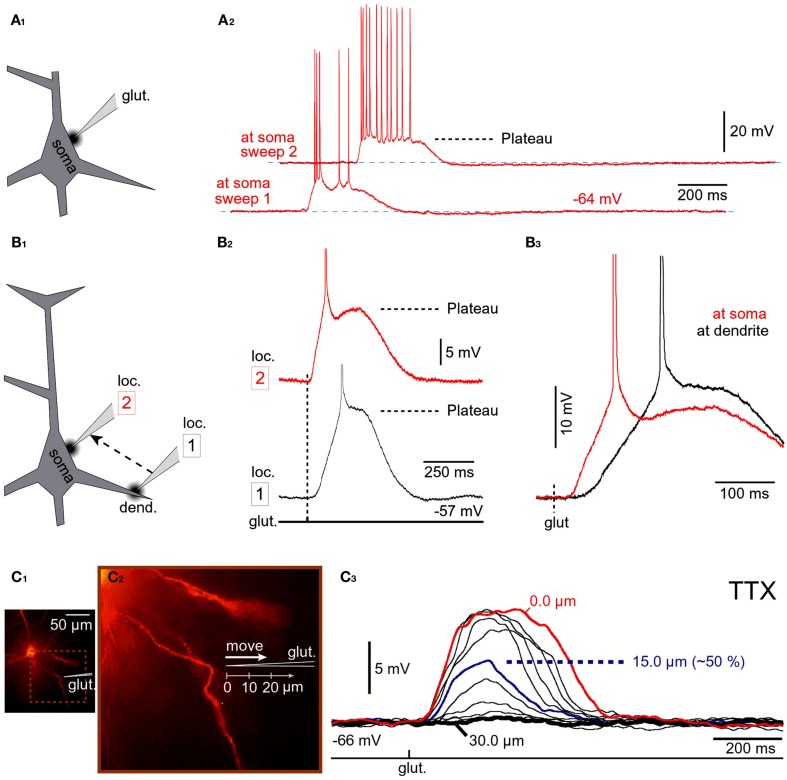
**Stimulation of NMDA receptors on the cell body**. **(A_1_)** Schematic representation of the experimental outline. The tip of the glutamate pipette is 10 μm from the soma. **(A_2_)** Neuronal response to somatic application of glutamate was tested using two intensities of iontophoretic glutamate current; pulse duration was fixed at 5 ms. **(B_1_)** Schematic representation of the experimental outline. The same glutamate-filled micropipette was alternated between dendritic (loc. 1) and somatic (loc. 2) stimulation sites. **(B_2_)** Glutamate-evoked plateau potentials at dendritic and somatic locations of the same cell; glutamate pulse unchanged. **(B_3_)** Same traces as in **(B_2_)** except superimposed on fast time scale. **(C_1_)** Microphotograph of a rhodamine-filled neuron. **(C_2_)** Schematic representation of the experimental outline. The tip of the glutamate application electrode (glut.) was gradually retracted in equal increments (3 μm). **(C_3_)** At each stop an identical glutamate pulse was applied in the presence of TTX (1 μM). At 15 μm away from the initial stimulation site, the amplitude of the glutamate-evoked depolarization is ~50% of its value at the initial stimulation site (0 μm). At 30 μm, glutamate is not detected by the dendrite.

The somatic and perisomatic membranes of cortical pyramidal neurons contain functional NMDA receptors (Dodt et al., 1998). Because there are no synaptic contacts or postsynaptic densities on the cell bodies and in the proximal 30 μm of basal dendrites (Larkman, 1991), all somatic and perisomatic NMDA receptors can be considered extrasynaptic. To test if activation of extrasynaptic NMDA receptors is sufficient for the initiation of glutamate-mediated plateau potentials, glutamate-filled micropipettes were positioned ~10 μm from the lateral edge of the cell body (Figure 6A_1_), also depicted in (Moore et al., 2011), their Figure 1. In all neurons treated this way (three out of three) glutamate pulses (5 ms duration) triggered plateau potentials lasting several hundred milliseconds (Figure 6A_2_). In order to compare the glutamate excitability of cell body and basal dendrites, in the next series of experiments plateau potentials were first evoked by glutamate microiontophoresis on basal dendrites (Figure 6B_1_, loc. 1). The same glutamate electrode was then positioned ~10 μm near the soma (Figure 6B_1_, loc. 2). In four neurons we kept the duration and intensity of glutamate iontophoretic pulse fixed at both locations, which allowed us to make quantitative comparisons. For the same glutamatergic stimulus, the ratio between somatic, and dendritic plateau was 0.91 ± 0.06 for amplitude and 0.106 ± 0.08 for duration (half-width), *n* = 17 measurements in four cells. In other words, 5-ms glutamate pulses were successful at maintaining the cell in a sustained depolarized state when applied on the cell body, as they were on spiny segments of basal dendrites (Figures 6B_2,B_3__). A major concern in these experiments was a potential spill of glutamate from the somatic stimulation site onto the neighboring dendrites. To determine how far glutamate can diffuse from the ejection site, the tips of glutamate iontophoretic electrodes were first positioned 5–10 μm from the shafts of visually identified basal dendrites (Figure 6C_1_), and the glutamate pipette was gradually retracted from the dendrite. Before the beginning of the pipette withdrawal procedure, the intensity of the glutamate iontophoretic current was adjusted so that a single 5-ms-long glutamate pulse triggered a somatic plateau potential (half-width, ~100 ms). With the iontophoretic current fixed at this value, the glutamate pipette was retracted in micrometer steps (Figure 6C_2_). At each stop, a glutamate pulse was re-applied and somatic response was recorded (Figure 6C_3_). At an average distance of 22.6 ± 2.5 μm (*n* = 13) the plateau potential was less than 50% of the initial value (at 0 μm). Since the initial 30 μm of basal dendrites are virtually spineless (Larkman, 1991; Ballesteros-Yanez et al., 2006), these data indicate that during somatic stimulations (Figures 6A,B) the dendritic spines were not involved in the initiation of plateau potentials. That is, the NMDA receptors located on the cell body and on the shafts of proximal dendritic segments (extrasynaptic NMDA receptors) can support glutamate plateau potentials.

### Persistent activity made from plateau potentials

**Figure 7:**
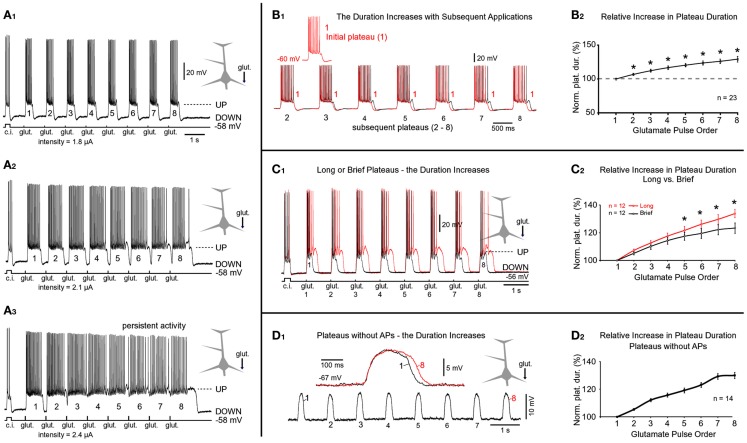
**Progressive increases in plateau durations during repetitive glutamatergic stimulations**. **(A_1_)** Glutamate pulses (duration = 5 ms) were delivered on the basal dendrite and evoked potentials were recorded in the soma. Prior to a train of eight glutamate pulses, the cell body was injected with depolarizing current to monitor neuronal intrinsic membrane excitability (current injection, c.i.). In subsequent sweeps **(A_2_,A_3_)**, the intensity of glutamate current was gradually increased, as indicated below the trace. **(B_1_)** The first plateau (*1*) is superimposed with each subsequent plateau in the train (2–8). **(B_2_)** Plateau half-widths (durations) were normalized against the first plateau in the train (*1*), averaged across 23 trains obtained in nine neurons, and plotted versus the chronological order of the glutamate pulse. *Paired *t*-test, *p* < 0.01, compared to the initial plateau *1*. [**(C_1_)***Red trace*: eight glutamate pulses; duration = 5 ms each] were delivered on one basal dendrite and evoked potentials were recorded in the soma. The stimulus intensity was then reduced to shorten the duration of plateaus by ~50% (black trace). **(C_2_)** *Red graph*: plateau durations were normalized against the first plateau in the sequence and averaged across 12 cells using traces with long plateaus (~500 ms). *Black graph*: traces with brief plateaus (~250 ms) from the same 12 cells were normalized and averaged. *p < 0.05, the corresponding plateaus were compared using paired *t*-test. **(D_1_)** Same experimental setting as in **(A_1_)** except plateau depolarizations did not trigger somatic action potentials. Inset: the first and the eighth plateaus are superimposed on a faster time scale. **(D_2_)** Plateau durations were normalized against the first plateau in the sequence and averaged across 14 cells using traces with 8 spikeless plateaus.

To investigate how temporal summation of glutamate-evoked plateau potentials may contribute to the neuronal AP output, glutamate-filled micropipettes were positioned in middle segments of basilar branches and glutamate was iontophoretically released for 5 ms, every second (eight glutamate pulses per train). In some experiments, the train of eight glutamate pulses was preceded by simple current injection into the soma (duration = 250 ms) to monitor neuronal integrity (Figure 7A_1_, c.i.). In these experiments, the starting intensity of glutamate stimulus was adjusted to trigger an event that was suprathreshold for both the dendrite (dendritic plateau potential) and the soma (somatic APs, Figures 3A,C, UP state). At lower glutamate stimulation intensities, each glutamate pulse produced an isolated event consisting of a sustained somatic depolarization accompanied by a burst of APs riding on top of the plateau depolarization (Figure 7A_1_). Between glutamate pulses the membrane potential promptly returned to the resting level (Figures 7A_1–A_3__). The rapid rise of the glutamate-evoked plateau potential and the rapid collapse at the end of its plateau phase, together with uniform amplitudes and durations of individual depolarizing events, are reminiscent of UP states that occur in cortical pyramidal neurons during slow wave sleep (Cowan and Wilson, 1994; Branchereau et al., 1996; Lewis and O’Donnell, 2000). Closer inspection of whole-cell recordings (Figure 7B_1_) as well as quantification of these data (Figure 7B_2_), revealed a tendency for the duration (but not the amplitude) to increase with the sequential order of the glutamate pulse. On average, each subsequent plateau was longer than the previous (Figure 7B_2_). Paired *t*-tests performed on the raw measurements before normalization detected significant differences between the first plateau (Figure 7B_1_, red) and each subsequent plateau in the train (Figure 7B_1_, black 2–8). The progressive increase in plateau half-width (Figure 7B_2_), especially prominent at higher glutamate stimulation intensities (Figures 7A_2,A_3__), suggests that residual glutamate was slowly increasing with repetitive glutamatergic input. The ability of glia to eliminate the focally released glutamate was diminished immediately following the first plateau (note the statistically significant difference between the *first* and the *second* plateau; Figure 7B_2_). Glutamate slowly accumulates in the extrasynaptic space on each following stimulus 2–8, resulting in a statistically significant difference between the first event and all subsequent events in a train (Figure 7B_2_, asterisks). These data suggested that the glutamate uptake and glutamate diffusion mechanisms failed to completely eliminate the residual glutamate.

### Residual glutamate

We next asked if stronger glutamatergic stimuli impose a greater burden on glutamate elimination than weaker stimuli. For this purpose, two trains consisting of eight glutamate pulses were delivered on the same dendrite. Following the first train (Figure 7C_1_, red trace), the intensity of iontophoretic current was decreased in order to reduce plateau duration by ~50% (Figure 7C_1_, black trace). The relative increases in plateau duration for traces with “long” plateaus (Figure 7C_1_, *red trace*) were significantly greater than for the traces with “brief” plateaus (Figure 7C_1_, *black trace*). Statistically significant differences were detected on the fifth, sixth, seventh, and eighth event in the train (Figure 7C_2_, *asterisks*). These data suggested that glutamate uptake and glutamate diffusion mechanisms are less effective in eliminating the residual glutamate for stronger glutamate stimulation intensities.

The previous experiments showed that the duration of glutamate-evoked plateau increases with subsequent applications of glutamate on basal dendrites. Does this phenomenon occur if the plateau was not accompanied by bursts of APs? To answer this question we applied glutamate trains (1 Hz) at distal dendritic segments (range 90–125 μm path distance from the soma, *n* = 14). At these stimulation sites, the dendritic plateau potentials often fail to trigger the somatic APs (Milojkovic et al., 2004; Major et al., 2008). In all neurons tested in this way (*n* = 14) we found a progressive increase in plateau duration (Figure 7D_1_). For example, the duration of plateau #8 (the eighth event in the sequence) was on average 130 ± 2.2% of the initial plateau (plateau #1, *n* = 14; Figure 7D_2_). This relative increase was very similar to the one obtained for plateaus accompanied by bursts of APs, 129 ± 4.1%; *n* = 23 (Figures 7B_1,B_2__). These data indicate that intrinsic membrane currents activated during AP burst firing are not responsible for the observed increase in plateau duration with subsequent glutamate applications.

### Fusion of plateau potentials

**Figure 8:**
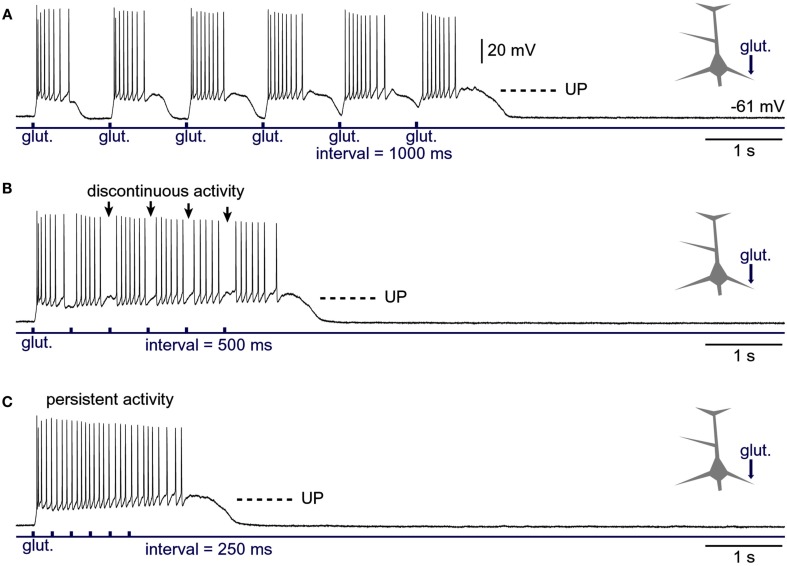
**Generation of persistent activity from consecutive plateau potentials**. **(A)** Six glutamate pulses (duration = 5 ms each) were delivered on the basal dendrite and evoked potentials were recorded in the soma. In subsequent sweeps **(B,C)**, the inter-pulse interval was gradually decreased, as indicated below the trace. Arrows in **(B)** mark pauses in AP firing.

In the previous experiments we varied the intensity of the glutamate stimulus while keeping the frequency of glutamate pulses fixed between trials (Figure 7A_1_). In the next series of experiments, we varied the interval between glutamate pulses in order to mimic increasing levels of cortical afferent activity arriving at the basilar dendritic tree. Decreasing the interval between glutamate pulses caused the duration of plateau potentials to increase with the sequential order of glutamate pulse in the train (Figure 8A), suggesting that residual glutamate slowly accumulates in the extracellular matrix surrounding NMDA receptors. Decreasing the interval between glutamate pulses caused plateau depolarizations to fuse into a continuous UP state (Figure 8B). In three out of three neurons tested in this way, the gradual fusion of glutamate plateau potentials (slow component) did not immediately result in a continuous train of APs. Instead, we observed apparent interruptions in AP firing in between individual glutamate pulses (Figure 8B, arrows), suggesting that pyramidal neurons can distinguish between bursts of electrical activity associated with individual glutamate-evoked dendritic plateau potentials even when their slow components are completely fused. However, this information became lost at higher stimulation frequencies, as further reduction in inter-pulse interval produced an uninterrupted train of APs (Figure 8C), reminiscent of persistent neuronal activity in the awake cortex (Timofeev et al., 2001).

### Spatial summation of plateau potentials

**Figure 9:**
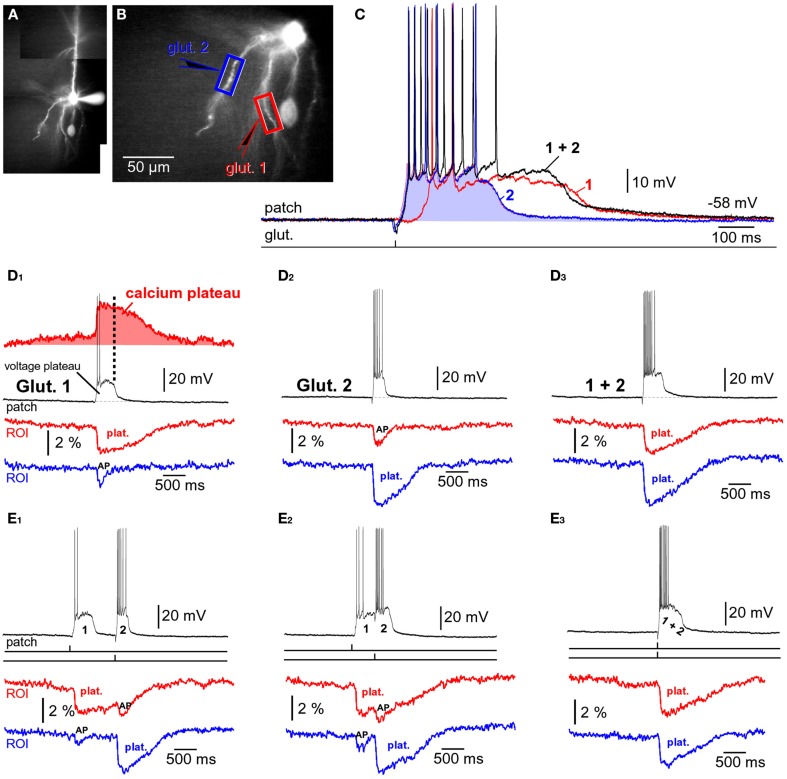
**Summation of glutamate-evoked plateau potentials**. **(A,B)** Pyramidal neuron filled with bis-fura-2 and Alexa Fluor 594. **(C)** Somatic membrane potential transients (patch) in response to one glutamate pulse (5 ms) delivered at location “*glut. 1*” (red trace), or one glutamate pulse delivered at location “*glut. 2*” (blue trace). Black trace (1 + 2) is the neuronal response to glut. 1 and glut. 2 pulses applied together. **(D_1_)** Simultaneous recordings of dendritic calcium transients at two regions of interest (ROIs) marked by boxes in **(B)**, and recording of somatic membrane potential (patch) during glutamate microiontophoresis at location “glut. 1.” “*Plat*.” marks Ca^2+^ plateau. “AP” marks dendritic calcium influx induced by backpropagating action potentials. Ca^2+^ signal from the red ROI was inverted, arbitrarily scaled, and the area underneath the trace shaded red. Vertical dashed line marks the end of the neuronal plateau depolarization. **(D_2_)** Same as in **(D_1_)**, except glutamate pulse was applied at location glut. 2. **(D_3_)** Same as in **(D_1_)**, except two pulses (1 and 2) were co-applied at the same moment. **(E_1_)** Ca^2+^ transients (ROIs) and somatic membrane potential (patch) during glutamate iontophoresis on two basal dendrites. Precise timings of glut. 1 and glut. 2 pulses are marked by vertical ticks beneath the patch recording. Time interval between glut. 1 and glut. 2 was 1000 ms. **(E_2_)** Same as in **(E_1_)**, except time interval between 1 and 2 was 500 ms. **(E_3_)** Same as in **(E_1_)**, except 1 and 2 were applied simultaneously (interval = 0 ms).

Layer 5 pyramidal neurons in the rat PFC typically contain three to eight primary basal dendrites and many more secondary and tertiary basal branches (Zhou et al., 2008, their Figure 10). With thousands of synaptic contacts distributed on the basilar dendritic tree (Larkman, 1991; Benavides-Piccione et al., 2006), it is likely that two or more basal dendrites can experience glutamate-dependent plateau potentials at the same moment of time. To study integration of dendritic plateau potentials, neurons were filled with fluorescent dyes (Figure 9A) and two glutamate-filled micropipettes were positioned in middle segments of two basal branches (Figure 9B). Glutamate was released for 5 ms from either micropipette (Figure 9C, *1* or *2*) and from both micropipettes at the same time (“*1* + *2*”). In order to determine the specificity of glutamate input onto one dendritic branch, experiments were initially performed in combination with simultaneous multi-site calcium imaging at 200 Hz frame rate (Antic, 2003). Every time an individual glutamate pulse resulted in somatic plateau depolarization, as detected in whole-cell recording (patch), there was an associated long-lasting calcium plateau at the glutamate input site; depicted in Figures 2D_1,D_2__. The duration of the dendritic calcium plateau was not determined by the duration of the corresponding voltage plateau, as calcium plateaus always persisted after the collapse of somatic plateau potential (Figure 9D_1_, vertical dashed line). Regardless of the stimulating electrode used (1 or 2), in each experimental trial the calcium plateau was restricted to one basal branch (Figures 9D_1,D_2__, plat.), indicating that focally applied glutamate did not diffuse from one dendrite to another. Unstimulated basal branches also experienced some calcium influx during plateau potentials; however, these calcium transients were smaller in amplitude and drastically shorter in duration (Figures 9D,E, AP). The peaks of smaller transients coincided with somatic APs in every sweep of every neuron tested in this way (*n* = 16 sweeps in three neurons). These calcium imaging data provide evidence that during paired (*1* + *2*) glutamate stimulations, the glutamate-evoked dendritic plateau potentials (Figure 2B) originate in two distinct branches (and no more) and summate in the cell body (Figure 9D_3_).

In the remainder of this paper we will quantify the summation of subthreshold and suprathreshold glutamate-evoked potentials arriving from two basal branches. All of the summands were active dendritic spikes – plateau potentials (suprathreshold in dendrite). The terms *sub-* and *supra-threshold* hereafter refer to the ability of an individual dendritic plateau potential to trigger somatic APs on its own; before the summation. An example of a subthreshold plateau is shown in Figure 7D_1_. An example of a suprathreshold plateau is shown in Figure 7B_1_.

### Spatial summation of subthreshold plateau potentials

**Figure 10:**
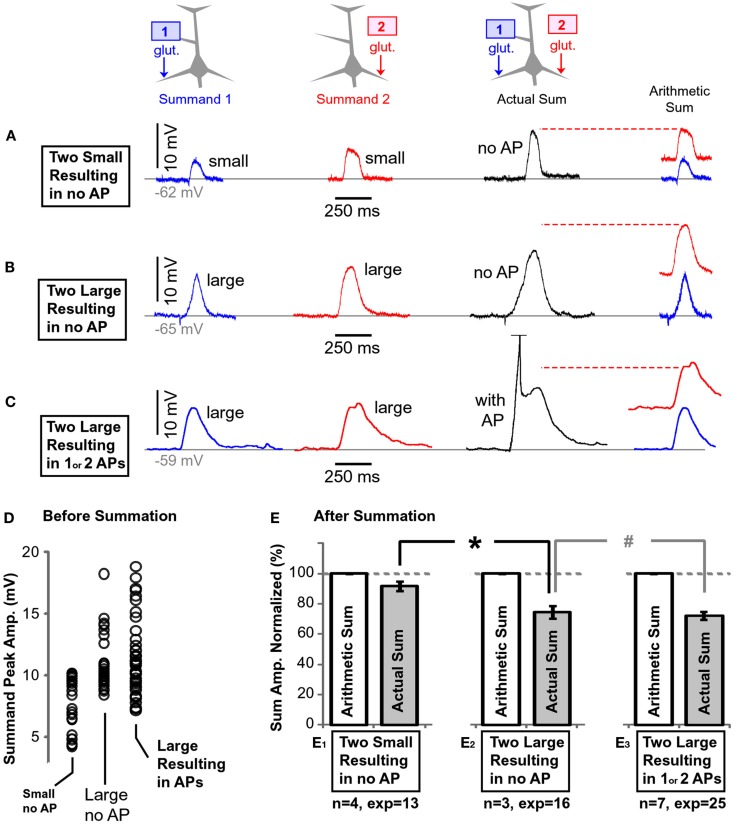
**Summation of plateau potentials in the absence of somatic AP firing**. **(A)** *Blue trace*: somatic recording of a plateau potential evoked by a 5-ms application of glutamate on the left basal branch, as schematically depicted by the drawing above the blue trace. *Red trace*: somatic recording of a plateau potential evoked by a 5-ms application of glutamate on the right basal branch, as schematically depicted in the drawing above the red trace. Note that each summand is “small,” as defined in Section “Materials and Methods.” *Black trace*: somatic depolarization resulting from glutamatergic stimulation of both dendrites at the same time, as depicted in the drawing above the black trace. *Arithmetic sum*: the dashed horizontal red line indicates the arithmetic sum of blue and red trace. **(B)** Same as in **(A)** except both summands (blue and red) are “large,” as defined in Section “Materials and Methods.” **(C)** Same as in **(B)** except summation resulted in AP firing (truncated). **(D)** Peak amplitudes of each summand (before summation) separated in three groups using criteria explained in Section “Materials and Methods.” **(E)** The amplitude of the actual depolarization (black trace) was divided by the corresponding arithmetic sum, and averaged across all experiments within one group. The name of the data group, number of cells (*n*) and the number of experiments (exp) are shown below each pair of bars. The dashed horizontal gray line marks the expected value if summation of two dendritic plateau potentials were perfectly linear. *p < 0.05 (unpaired *t*-test). #*p* > 0.05.

To study the integration of subthreshold plateau potentials, neurons were filled with fluorescent dyes and two glutamate-filled micropipettes were positioned in middle segments of two basal branches (Figure 9B). Glutamate was released for 5 ms from either micropipette (*1* or *2*) and from both micropipettes at the same time (“*1* + *2*”), as depicted schematically by three drawings on the top of Figure 10. Based on the amplitudes of individual plateau potentials before summation (“Summand *1*” or “Summand *2*”), the experimental data was divided into 3 groups: “*Two Small Resulting in no AP*”; “*Two Large Resulting in no AP*;” and “*Two Large Resulting in 1 or 2 APs*.”

When both summands (“*1*” or “*2*”) were small (as defined in Materials and Methods), their physiological sum in the cell body was similar in amplitude to their arithmetic sum (Figure 10A); the summation was linear. Thirteen such experiments carried out in four neurons were quantified, normalized against their corresponding arithmetic sums, averaged, and displayed in the bar diagram (Figure 10E_1_). The average summand amplitude in the “Small no AP” group was 7.4 ± 0.35 mV (*n* = 26 summands in 13 pairs). The distribution of the plateau amplitudes before summation is plotted in (Figure 10D, *Small no AP*).

When both summands were large (as defined in the Materials and Methods), their physiological sum in the cell body was less than their arithmetic sum (Figure 10B). Sixteen experiments from three neurons were quantified and displayed in the bar diagram (Figure 10E_2_). Because the actual sum was considerably less than the arithmetic sum (Figure 10E_2_), the summation process was characterized as “sublinear.” The summation process in group “Large no AP” was inherently different from the summation process in group “Small no AP”; confirmed by a comparison of the relative amplitudes after normalization (Figure 10E, asterisk, unpaired, *p* < 0.01). The average amplitude of all summands in the “Large no AP” group was 10.8 ± 0.39 mV (*n* = 32 summands). The distribution of the summand amplitudes in this group is plotted in (Figure 10D, *Large no AP*).

If both summands were sufficiently large so that their summation resulted in the somatic plateau depolarization accompanied by 1 or 2 APs, again the amplitude of the actual slow component in the cell body was less than the arithmetic sum of two individual dendritic plateaus (sublinear summation; Figure 10C). Twenty-five such experiments from seven neurons were quantified and displayed in the bar diagram (Figure 10E_3_). The average summand amplitude in this group was 11.3 ± 0.44 mV (*n* = 50 summands, Figure 10D, *Large Resulting in APs*).

In summary, these data show that “small” plateau potentials (both small, see Materials and Methods) summate linearly in the cell body (Figure 10E_1_). Larger plateaus, on the other hand, summate sublinearly – their physiological sum is always less than expected from a simple arithmetic summation (Figure 10E_2_). The presence of somatic AP firing did not change the outcome of summation, as determined by unpaired *t*-test (Figure 10E_3_, “#,” *p* > 0.05).

### Spatial summation of suprathreshold plateau potentials

**Figure 11:**
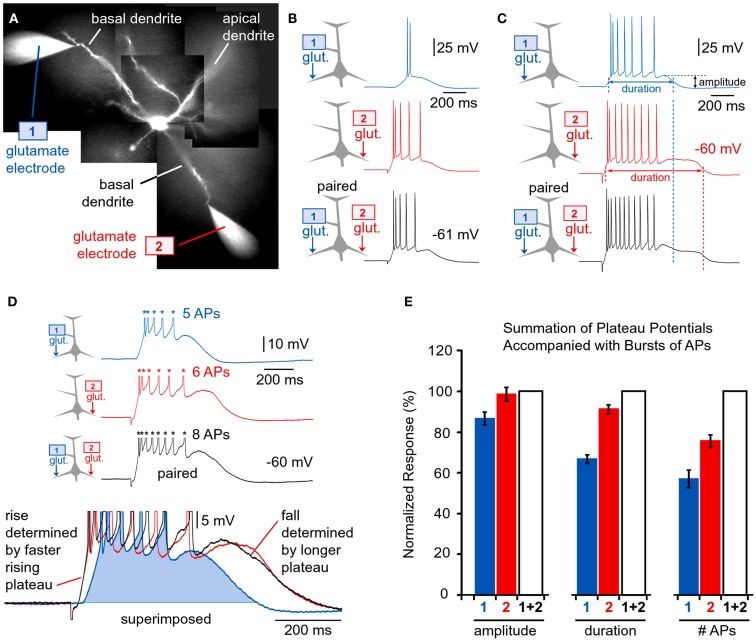
**Quenching of dendritic plateau potentials at the soma**. **(A)** Two rhodamine-filled glutamate electrodes positioned next to two rhodamine-filled basal dendrites. **(B–D)** Drawing of an experimental outline precedes each whole-cell recording. Whole-cell recordings are colored to match the glutamate pipette used. Black traces indicate simultaneous release from both glutamate electrodes (paired). Vertical dashed line marks the end of the corresponding plateau. **(D)** Inset: glutamate-evoked plateau potentials in response to *1*, *2*, and *1* + *2* stimulation are superimposed to show that the shorter plateau potential (blue, *glut. 1*) is totally eclipsed by the longer plateau potential (red, *glut. 2*) during co-activation (black, *1* + *2*, paired). **(E)** Amplitude of the glutamate-evoked depolarization [slow component amplitude marked in **(C)**] is divided by the plateau amplitude obtained during co-activation (*1* + *2*), and averaged across nine neurons; 21 triplets of sweeps. Duration of the plateau depolarization was measured at half amplitude [as indicated in **(C)**], normalized against *1* + *2* and averaged across 21 triplets of sweeps obtained in nine neurons. The number of action potentials (#AP) was normalized against trace “*1* + *2*” and averaged across seven neurons. Plateau potentials of smaller amplitude and shorter duration (blue traces) are completely eclipsed by larger and longer plateaus (red traces), when paired (black traces).

The parameters of glutamatergic inputs (dendritic location, iontophoretic current intensity, pulse duration) were adjusted so that each dendritic potential by itself brings the cell body into an UP state crowned with AP firing (e.g., Figure 7B_1_). When two such dendritic UP states collide in the cell body (Figure 11B, black trace), the amplitude of somatic plateau depolarization (plateau amplitude as defined in the Materials and Methods) is not greater than the individual components, glut. 1 or glut. 2 (Figure 11B, blue and red trace). When two dendritic UP states collide in the cell body, the duration of the resulting somatic plateau depolarization (plateau duration) is not greater than the duration of the longer individual component. Compare the durations of somatic plateaus in red (glut. 2 only) and black (paired) traces in three experiments shown in Figures 11B–D. The only somatic parameter that seemed to reflect the co-occurrence of two suprathreshold dendritic plateau potentials in the basilar dendritic tree was the frequency of AP firing. The cell bodies of layer 5 pyramidal neurons fired a greater number of APs during the simultaneous occurrence of two plateaus than during each dendritic plateau potential alone (Figure 11D). However, the summation of spike numbers was still sublinear. For example, a five-spike potential (in response to glut. 1) and six-spike potential (glut. 2), when triggered together generated eight spikes (Figure 11D, black trace), which is only 75% of what should be expected if spike numbers were additive biophysical parameters (5 + 6 = 11). Two individual plateau potentials coming from two basal branches at the same moment of time integrate in such a manner that the number of APs generated per plateau is greater than the number generated from either individual component on its own (Figure 11E, #APs). Quantification of data obtained in nine pyramidal neurons (Figure 11E) was performed by normalizing plateau amplitudes, plateau durations, and spike numbers per plateau (*glut. 1* or *glut. 2*) against values obtained when *1* and *2* were paired. The numbers were arranged so that smaller amplitudes, shorter durations, and fewer APs per plateau were always named *glut. 1* (Figure 11E, blue columns), and larger ones were always named *glut. 2* (Figure 11E, red columns). This was done to emphasize that smaller and shorter plateau potentials (Figure 11D, Inset, blue trace) are eclipsed by larger and longer plateaus (red trace), when paired together (black trace). In other words, from the point of the somatic voltage change (i.e., slow component), a smaller or shorter plateau potential is completely invisible if co-occurring with a larger and longer plateau potential. It must be noted that this conclusion applies only if two dendritic plateaus are perfectly aligned in time. If one plateau begins sooner or ends later than the plateau arriving from the other basal dendrite, these timing discrepancies will be detected in the somatic voltage waveform. The average duration of paired stimulations, “1 + 2” (Figure 11E, duration, white column) was slightly greater than the duration of the individual (longer) plateau in the pair (“2,” red column). This is because in some experiments the rise of the shorter plateau (blue column) was faster than the rise of the longer plateau (red column), as shown in Figure 9C (the rise of the blue trace precedes the rise of the red trace). When two individual plateau potentials coming from two basal branches are not perfectly synchronized, then they integrate in such a way that the cell body is maintained in a depolarized UP state for a longer period of time than either individual component would do on its own (Figure 9E_2_). Although it may appear from the graph in Figure 11E that both plateau duration and the AP count increase by summation, these gains are made for different reasons. The gains in duration (Figure 11E, duration) only occur if the summating plateau potentials are not perfectly synchronous. The gains in the number of APs (Figure 11E, #APs) occur regardless of whether or not the two summating plateaus are perfectly synchronous in rise and decline.

## Discussion

The experiments based on two-photon imaging of a spine and its adjacent dendritic shaft unequivocally showed that extrasynaptic NMDA receptors are activated during synaptically evoked NMDA spikes (Chalifoux and Carter, 2011). If two synaptic inputs at 50 Hz (Figure 2A) activated extrasynaptic receptors via glutamate spillover (Chalifoux and Carter, 2011), then five of such synaptic inputs would cause even stronger activation of extrasynaptic NMDA receptors, as the amount of glutamate released by five shocks exceeds that released by two synaptic shocks. Five synaptic stimulations produced dendritic plateau potentials (Figure 2B). Therefore, dendritic NMDA spikes (Polsky et al., 2004; Chalifoux and Carter, 2011) and dendritic plateau potentials (Milojkovic et al., 2004, 2005a) represent two characteristic physiological states involving the activation of extrasynaptic NMDA receptors. Although their cellular mechanisms are essentially the same: (1) clustered glutamatergic inputs in space and time (Larkum and Nevian, 2008; Magee, 2011), (2) failure of glial processes to clear the surplus of glutamate (Suzuki et al., 2008; Chalifoux and Carter, 2011), and (3) activation of extrasynaptic NMDA receptors (Tovar and Westbrook, 1999; Harris and Pettit, 2007), these two signals (NMDA spike and plateau potential) still represent two qualitatively different events in dendritic integration (Figures 2A,B).

Our emphasis on physiological differences between NMDA spikes (Polsky et al., 2004; Chalifoux and Carter, 2011) and plateau potentials (Wei et al., 2001; Milojkovic et al., 2004, 2005a; Suzuki et al., 2008) are based on side-by-side comparison of these two potentials in the basal dendrites of L5 pyramidal neurons in the rat PFC. We have shown that dendritic NMDA spikes and dendritic plateau potentials differ by two fundamental physiological properties: (1) voltage waveform and (2) calcium influx. The NMDA spikes are predominantly subthreshold events at the cell body that resemble pointy EPSPs (Figure 2A, Inset). Synaptically evoked dendritic plateau potentials are endowed with much larger depolarization amplitudes that reach the threshold for AP firing (Figure 2B). Furthermore, plateau potentials last much longer than NMDA spikes, providing stable depolarizations of the cell body for several hundred milliseconds. The calcium influx associated with an NMDA spike is restricted to a very narrow dendritic segment; ~20 μm in length (Figure 2C). A dendritic plateau potential, in contrast, triggers a massive calcium influx at the glutamate input site and very substantial calcium signals in the remainder of the dendrite (Figure 2D). The massive calcium plateau at the input site is mediated by NMDA receptor channels (Milojkovic et al., 2007; Major et al., 2008), while calcium signals in the remainder of the input receiving dendrite are mediated by bi-directional propagation of the dendritic plateau potential (Milojkovic et al., 2004, 2005a, 2007). Overall, dendritic NMDA spikes and dendritic plateau potentials are two distinct outcomes of dendritic processing of glutamatergic inputs in thin dendrites of cortical pyramidal neurons, potentially having distinct functional roles in dendritic plasticity, synaptic plasticity, and information processing (Antic et al., 2010). For example, slow dendritic dynamics are necessary for the stabilization of network activity bumps in noisy networks (Morita, 2008; Kurashige and Cateau, 2011). “Slow dendritic dynamics” is embodied in the plateau phase of the glutamate-mediated plateau potential, while the voltage waveform of the NMDA spike clearly lacks this plateau phase (Figure 2A, blue trace).

### Glutamate pond

Dendritic NMDA spikes were thought to be regenerative membrane potentials (spikes) carried by the negative slope conductance of the NMDA receptor current (Mayer et al., 1984; Nowak et al., 1984). The experimental proof of dendritic regenerative properties was based on somatic recordings (Schiller et al., 2000; Major et al., 2008). We have shown that the dendritic membrane potential actually exhibits the characteristic transition from subthreshold potential to spike in the presence of TTX (Milojkovic et al., 2005a), their Figure 8) and in drug-free saline (Figure 3A, ROI). At the same instant of time when a basal dendrite jumps from a subthreshold to a plateau potential, the somatic membrane potential invariably jumps from subthreshold to plateau potential (Figure 3A, brown trace). The slow component of the somatic voltage waveform of cortical pyramidal neurons is nothing more than a reflection of the glutamatergic integration event occurring somewhere in the dendritic tree (Milojkovic et al., 2005a; Antic et al., 2007). Simply, the cell body is in DOWN state if the dendrite is in DOWN state. When the dendrite transitions from DOWN to UP state, the cell body passively follows, by transitioning from DOWN to UP state (Figures 3A,B). The ability of thin dendritic branches to deliver sustained depolarizing current to the soma was firmly established in experiments that combined somatic recordings with dendritic imaging (Oakley et al., 2001; Wei et al., 2001; Cai et al., 2004; Milojkovic et al., 2004; Major et al., 2008). This mechanism may be involved in the cellular manifestation of cortical UP states during slow wave sleep (Milojkovic et al., 2004, 2005a; Antic et al., 2007). Dendritic plateau potentials are not causing cortical UP states – they are just reporting network UP states to the neuronal cell body (Antic et al., 2010). Initial efforts to detect dendritic plateau potentials *in vivo* have failed (Waters and Helmchen, 2004; Jia et al., 2010), but recently some experimental data has begun to suggest that clustered synaptic activity and highly localized calcium influxes potentially associated with local dendritic spikes may occur spontaneously in cortical networks (Katona et al., 2011; Kleindienst et al., 2011; Varga et al., 2011).

Although dendritic NMDA spikes have been shown to exhibit a voltage threshold (Schiller et al., 2000; Milojkovic et al., 2004; Rhodes, 2006), recent experimental evidence of combined glutamate-voltage sensitivity (Major et al., 2008; Polsky et al., 2009) sheds new light on this biophysical phenomenon. The spike mechanism is complex because the presence of glutamate molecules is not simply a permissive factor, but it may also represent the primary mechanism of the observed voltage jump from subthreshold to spike (Schiller et al., 2000; Milojkovic et al., 2004, 2005a; Cha-Min Tang, personal communication). Several lines of evidence indicate that glutamate plays a more important role than voltage. For example, the experimental finding that spike-mediated calcium influx is severely restricted to a narrow, ~20 μm segment of the basal branch (Figure 2C_1_) emphasizes an overriding importance of reaching the NMDA conductance threshold locally within a dendritic branch, as opposed to simply reaching a local voltage threshold (Polsky et al., 2009). In the two-pulse stimulation paradigm (Figure 2A), the carryover of depolarization from the first pulse in a pair had surprisingly little impact on the stimulus threshold at which an NMDA spike could be generated by a second pulse (Polsky et al., 2009). That is, dendritic voltage plays a relatively minor role in setting a better-or-worse initial condition for NMDA spike generation (Polsky et al., 2009). Here we postulate that the glutamate threshold (Milojkovic et al., 2005b; Major et al., 2008; Polsky et al., 2009) is reached when glia can no longer cope with repetitive glutamatergic inputs arriving in a confined space (Figure 1B). For a brief period of time the dendritic segment is surrounded by a surplus of glutamate (Figure 1C, “glutamate pond”). During such an overwhelming glutamatergic stimulus, the dendritic spike cannot be perturbed by voltage (Figures 5B,C; Major et al., 2008). The finding that NMDA receptors on the cell body and on the most proximal dendritic segments (extrasynaptic receptors by definition) can readily support plateau potentials (Figure 6) is consistent with the idea that extrasynaptic NMDA receptors are the major carriers of the plateau current during dendritic plateau potentials (Suzuki et al., 2008), and also that the dendritic shaft is surrounded by an abundance of glutamate during that period of time (Figure 1C). The NMDA spike initiation is sensitive to the frequency of synaptic activation (Polsky et al., 2004, 2009) not exclusively because of the temporal summation of voltage, but primarily due to a chemical summation of the glutamate originating from two sources: (1) synaptic spillover and (2) release from astrocytic processes (Parpura et al., 1994; Jourdain et al., 2007; Min and Nevian, 2012).

The well-established requirement for the initiation of NMDA spikes is to shock synaptic terminals more than once (Polsky et al., 2004; Chalifoux and Carter, 2011; Figure 2A). These recent publications suggest that repetitive input is not needed to depolarize the dendrite to some voltage threshold for spike initiation, but instead multiple shocks are necessary to reverse glial function from glutamate uptake to glutamate release (Figure 1C, dark green glia). In glutamate uncaging experiments, the NMDA spike is initiated only when experimenters select neighboring dendritic spines (Losonczy et al., 2008; Remy et al., 2009; Branco and Hausser, 2011). The new evidence (Major et al., 2008; Polsky et al., 2009, present data) suggests that dendritic spikes in glutamate uncaging experiments do not arise from summation of voltage alone, but rather from summation of three glutamate sources: (1) uncaged glutamate; (2) synaptically released glutamate triggered by the presence of uncaged glutamate; and (3) glutamate released from glia (Min and Nevian, 2012) stimulated by uncaged glutamate.

### Temporal summation of suprathreshold plateau potentials

Early experiments employed temporal summation of *subthreshold* depolarizations (Milojkovic et al., 2004, 2007; Polsky et al., 2004, 2009). In the present project we investigated neuronal responses to temporal summation of *suprathreshold* dendritic plateau potentials. We sought to investigate the somatic voltage response during a period of time when a narrow segment of a basal dendrite is bombarded with suprathreshold glutamatergic inputs; suprathreshold both in the dendrite (dendritic spike) and in the soma (AP; Figure 3A). Instead of going into depolarization blocks from overwhelming excitatory input (Campbell and Hablitz, 2005), PFC L5 pyramidal neurons alternated from a resting (DOWN) to a depolarized (UP) state, with each given pulse, thus faithfully reporting each glutamatergic salvo at low frequency (~1 Hz, Figure 7A_1_). These data indicate that basal dendrites of PFC L5 pyramidal neurons are endowed with a robust membrane mechanism, capable of decoding large amounts of excitatory neurotransmitter into sustained depolarizations and then quickly recovering from the activation of ligand-gated and voltage-gated dendritic conductances. Suprathreshold salvoes of glutamate, arriving at frequencies higher than 1 Hz, also showed no signs of a depolarization block. Instead, individual plateaus gradually merged into one continuous depolarized state (Figure 8C). Sustained depolarizations with persistent AP firing that are occasionally interrupted by a DOWN state (Figure 7A_3_), are reminiscent of intracellular recordings obtained in animal cortices during transitions from sleep to awake state (Timofeev et al., 2001). According to our working model, the interruptions in persistent activity (transient DOWN states, Timofeev et al., 2001) indicate brief periods of time in which the instantaneous count of plateau potentials across the entire basilar dendritic tree was equal to zero.

### Spatial summation of suprathreshold plateau potentials

In the elaborate dendritic tree of one cortical pyramidal neuron, substantial glutamatergic inputs impinge simultaneously on two or more dendritic branches (Varga et al., 2011), providing the necessary conditions for spatial summation of dendritic voltages in the cell body. Summation of subthreshold synaptic inputs has been initially studied in multicompartmental biophysical models (London and Segev, 2001; Poirazi et al., 2003; Polsky et al., 2009). In brain slice experiments the summation of subthreshold potentials was explored using two glutamate iontophoresis electrodes (Cash and Yuste, 1999), or dual pipette synaptic stimulation (Polsky et al., 2004; Larkum et al., 2009). The traditional view of signal integration in the cerebral cortex is based on the idea that the neuronal computational task is to summate thousands of subthreshold synaptic inputs (Cash and Yuste, 1999; London and Segev, 2001). Departing from the traditional, in the present study we asked what the neuronal response would be if two dendritic plateau potentials, each capable of bringing the cell body into firing a burst of APs on its own, occurred at two dendritic locations simultaneously. We studied the summation of glutamatergic inputs that were pre-integrated into robust local spikes at their respective dendritic integration sites, consistent with a two-stage model of pyramidal neuron, where the first stage of signal integration takes place in the dendrite (Poirazi et al., 2003). In our experimental design, the individual elements of summation were not only suprathreshold for the dendrite (dendritic plateau-spike) but also for the cell body (plateau depolarization crowned by bursts of APs, Figures 9D_1,D_2__). Surprisingly, robust glutamate-evoked plateau potentials arriving from two basal branches into the cell body, each contributing ~20 mV of depolarization on its own, did not contribute to the amplitude of the slow component of somatic depolarization when co-applied at the same moment of time (Figure 11E, *amplitude*). Additionally, two robust plateau potentials, each lasting hundreds of milliseconds on its own, did not contribute to the duration of somatic plateau depolarization when arriving at the same instance of time. A longer plateau regularly eclipsed the shorter plateau (Figure 11D, inset). The only way for shorter plateaus to affect the somatic voltage was to start before, or end after the longer plateau (Figure 9C). These results (Figures 9–11) may explain why the summation of dendritic plateau potentials was never suspected from intracellular recordings (Timofeev et al., 2001). The “quenching” of robust suprathreshold events in the same unit of time (Figure 11E, *amplitude*) explains why, in a given neuron, all cortical UP states have uniform amplitudes (Cowan and Wilson, 1994; Branchereau et al., 1996; Lewis and O’Donnell, 2000; Timofeev et al., 2001). Two or more suprathreshold dendritic plateau potentials, arriving from two basal dendrites at the same moment of time, produce similar amplitude of sustained depolarization as either plateau alone (Figure 11E, amplitude).

### Signal comppression in cortical pyramidal neurons

**Figure 12:**
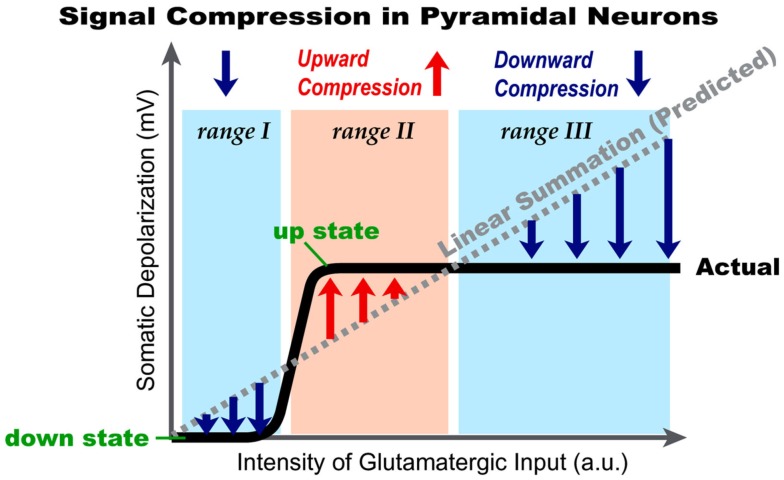
**Cellular bases of signal compression in the basilar dendritic tree of cortical pyramidal neurons**. Schematic representation of data obtained by summation of glutamatergic inputs in the basilar dendritic tree. Thick black line (*Actual*) represents a sigmoidal shape of somatic depolarization obtained experimentally by gradually increasing the intensity of glutamatergic stimulation delivered on one basal dendrite or by summation of inputs from two basal branches. The dashed gray line represents an expected depolarization in the cell body, based on the arithmetic (linear) summation of plateau potentials occurring in one or two basal dendrites simultaneously. Pink rectangle marks the range of glutamatergic input intensities (*range II*), in which cellular (dendritic) mechanisms boost the amplitude of the somatic depolarization. Blue rectangle marks the range of glutamatergic input intensities (*range III*), in which cellular mechanisms (dendritic and somatic) suppress the amount of somatic depolarization. As a result of active boosting (red arrows) or active suppression (blue arrows) the neuronal output (somatic depolarization) is compressed in a narrow range of amplitudes, marked by “*up state*.”

The extrasynaptic NMDA receptors and voltage-gated K^+^ currents together provide pyramidal neurons with the ability to respond to a wide range of input intensities (Figure 12, *ranges II and III*) with a very narrow range of somatic depolarizations (Figure 12, UP state). This function is analogous to a dynamic range compression in audio engineering. Audio compressors amplify quiet sounds (*upward compression*) and limit or reduce the volume of loud sounds (*downward compression*). In our working model (Figure 12), the quiet sounds are equivalent to weak glutamatergic inputs; and the loud sounds are equivalent to suprathreshold glutamatergic inputs. If a pyramidal neuron were a simple integrator, the somatic depolarization would be proportional to the intensity of glutamatergic input received by dendrites (Figure 12, dashed gray line). However, our experimental measurements (Figures 3E and 4D) indicate that the amplitude of the somatic depolarization remains in a very narrow range of values despite the increasing intensity of glutamatergic drive (Figure 12, abscissa). In one particular range of glutamatergic intensities (Figure 12, *range II*) the cellular mechanisms boost the amplitude of the somatic depolarization (Figure 12, *Upward compression*, red arrows). In another range of glutamatergic intensities (Figure 12, *range III*) the cellular mechanisms work together to suppress the amplitude of the somatic depolarization (Figure 12, *Downward compression*, blue arrows). *UPWARD COMPRESSION*: excitatory inputs that reach the local threshold are amplified by regenerative properties of NMDA currents in basal dendrites (Schiller et al., 2000; Milojkovic et al., 2004, 2005a; Rhodes, 2006; Major et al., 2008) to a small degree, and by local summation of released glutamate in a confined extracellular space to a larger degree (Figure 1C). Glutamate required for reaching the glutamate threshold (Major et al., 2008; Polsky et al., 2009) is not only released from the active synaptic afferents (Figure 1C), but also from synaptically activated astrocytic processes (Parpura et al., 1994; Jourdain et al., 2007; Min and Nevian, 2012). Both the dendritic membrane currents and the accumulation of glutamate in the extracellular space effectively boost near-threshold afferent inputs, causing the neuronal output function to shift upward (Figure 12, red arrows). *DOWNWARD COMPRESSION*: glutamatergic inputs that greatly exceed the threshold for the creation of a local glutamate pond cannot depolarize the dendritic membrane above the glutamate reversal potential (~0 mV). In addition to this hard limit, the dendritic K^+^ currents activated by the voltage waveform of dendritic plateau potentials (Milojkovic et al., 2004, 2005a) and by a massive calcium influx (Milojkovic et al., 2007; Major et al., 2008) limit the amount of dendritic depolarization (Wilson and Kawaguchi, 1996; Contreras et al., 1997; Cai et al., 2004). These dendritic plateau potentials are then attenuated by dendritic cable properties en route to the soma (Milojkovic et al., 2004; their Figures 4 and 7) and finally compressed by AP-activated K^+^ current in the somatic membrane (Bekkers, 2000; Korngreen and Sakmann, 2000; Schaefer et al., 2007). Note that plateau amplitudes increased upon blocking APs with TTX (Figure 4B). Because of these biophysical properties, the maximal amount of depolarizing current that basal dendrites can generate and deliver to the cell body is strictly limited. Once the dendritic plateau potential has been initiated, an entire range of suprathreshold glutamatergic inputs (Figure 12, *ranges II and III*) actually produces only one current amplitude (Figure 12, *UP state*). That is, during a dendritic UP state (Figures 2B, 3A, 4C_2_, and 7A) mediated by extrasynaptic NMDA receptors (Suzuki et al., 2008; Chalifoux and Carter, 2011), the neuronal output is strongly compressed in a narrow range, fluctuating around a single value (Figure 12, *UP state*). Dynamic range compression manifested by a steep sigmoidal input-output function (Figure 12, *Actual*) has been implicated as one of the crucial components of signal processing in the central nervous system (Geisler and Albrecht, 1995; Clatworthy et al., 2003; Nizami, 2005; Persi et al., 2011).

## Author Contributions

The work was done in Srdjan D. Antic laboratory. Srdjan D. Antic designed the experiments. Katerina D. Oikonomou, Shaina M. Short, Matthew T. Rich, and Srdjan D. Antic performed the experiments and analyzed the data. Manuscript was drafted by Srdjan D. Antic. Comments on drafts and approval of the final version: all authors.

## Conflict of Interest Statement

The authors declare that the research was conducted in the absence of any commercial or financial relationships that could be construed as a potential conflict of interest.

## References

[B1] Acker C. D. Antic S. D. (2009). Quantitative assessment of the distributions of membrane conductances involved in action potential backpropagation along Basal dendrites. J. Neurophysiol. 101, 1524–1541 10.1152/jn.00651.2007 19118105 PMC2666409

[B2] Antic S. Major G. Zecevic D. (1999). Fast optical recordings of membrane potential changes from dendrites of pyramidal neurons. J. Neurophysiol. 82, 1615–1621 10482775 10.1152/jn.1999.82.3.1615

[B3] Antic S. D. (2003). Action potentials in basal and oblique dendrites of rat neocortical pyramidal neurons. J. Physiol. (Lond.) 550, 35–50 10.1113/jphysiol.2002.033746 12730348 PMC2343022

[B4] Antic S. D. Acker C. D. Zhou W. L. Moore A. R. Milojkovic B. A. (2007). “The role of dendrites in the maintenance of the UP state,” in Mechanisms of Spontaneous Active States in the Neocortex, ed. Timofeev I. (Kerala: Research Signpost), 45–72

[B5] Antic S. D. Zhou W. L. Moore A. R. Short S. M. Ikonomu K. D. (2010). The decade of the dendritic NMDA spike. J. Neurosci. Res. 88, 2991–3001 10.1002/jnr.22444 20544831 PMC5643072

[B6] Ariav G. Polsky A. Schiller J. (2003). Submillisecond precision of the input-output transformation function mediated by fast sodium dendritic spikes in basal dendrites of CA1 pyramidal neurons. J. Neurosci. 23, 7750–7758 12944503 10.1523/JNEUROSCI.23-21-07750.2003 PMC6740608

[B7] Asztely F. Erdemli G. Kullmann D. M. (1997). Extrasynaptic glutamate spillover in the hippocampus: dependence on temperature and the role of active glutamate uptake. Neuron 18, 281–293 10.1016/S0896-6273(00)80268-8 9052798

[B8] Ballesteros-Yanez I. Benavides-Piccione R. Elston G. N. Yuste R. DeFelipe J. (2006). Density and morphology of dendritic spines in mouse neocortex. Neuroscience 138, 403–409 10.1016/j.neuroscience.2005.11.038 16457955

[B9] Bekkers J. M. (2000). Properties of voltage-gated potassium currents in nucleated patches from large layer 5 cortical pyramidal neurons of the rat. J. Physiol. 3, 593–609 10.1111/j.1469-7793.2000.t01-1-00593.x 10856115 PMC2269964

[B10] Benavides-Piccione R. Hamzei-Sichani F. Ballesteros-Yanez I. DeFelipe J. Yuste R. (2006). Dendritic size of pyramidal neurons differs among mouse cortical regions. Cereb. Cortex 16, 990–1001 10.1093/cercor/bhj041 16195469

[B11] Benucci A. Verschure P. F. Konig P. (2004). Two-state membrane potential fluctuations driven by weak pairwise correlations. Neural Comput. 16, 2351–2378 10.1162/0899766041941871 15476604

[B12] Branchereau P. Van Bockstaele E. J. Chan J. Pickel V. M. (1996). Pyramidal neurons in rat prefrontal cortex show a complex synaptic response to single electrical stimulation of the locus coeruleus region: evidence for antidromic activation and GABAergic inhibition using in vivo intracellular recording and electron microscopy. Synapse 22, 313–331 10.1002/(SICI)1098-2396(199604)22:4<313::AID-SYN3>3.3.CO;2-N 8867026

[B13] Branco T. Hausser M. (2011). Synaptic integration gradients in single cortical pyramidal cell dendrites. Neuron 69, 885–892 10.1016/j.neuron.2011.02.006 21382549 PMC6420135

[B14] Cai X. Liang C. W. Muralidharan S. Kao J. P. Tang C. M. Thompson S. M. (2004). Unique roles of SK and Kv4.2 potassium channels in dendritic integration. Neuron 44, 351–364 10.1016/j.neuron.2004.09.026 15473972

[B15] Campbell S. Hablitz J. J. (2005). Modification of epileptiform discharges in neocortical neurons following glutamate uptake inhibition. Epilepsia 46(Suppl. 5), 129–133 10.1111/j.1528-1167.2005.01020.x 15987267

[B16] Cash S. Yuste R. (1999). Linear summation of excitatory inputs by CA1 pyramidal neurons. Neuron 22, 383–394 10.1016/S0896-6273(00)81098-3 10069343

[B17] Chalifoux J. R. Carter A. G. (2011). Glutamate spillover promotes the generation of NMDA spikes. J. Neurosci. 31, 16435–16446 10.1523/JNEUROSCI.4561-10.2011 22072693 PMC3235338

[B18] Clatworthy P. L. Chirimuuta M. Lauritzen J. S. Tolhurst D. J. (2003). Coding of the contrasts in natural images by populations of neurons in primary visual cortex (V1). Vision Res. 43, 1983–2001 10.1016/S0042-6989(03)00277-3 12831760

[B19] Contreras D. Durmuller N. Steriade M. (1997). Plateau potentials in cat neocortical association cells in vivo: synaptic control of dendritic excitability. Eur. J. Neurosci. 9, 2588–2595 10.1111/j.1460-9568.1997.tb01688.x 9517464

[B20] Cowan R. L. Wilson C. J. (1994). Spontaneous firing patterns and axonal projections of single corticostriatal neurons in the rat medial agranular cortex. J. Neurophysiol. 71, 17–32 8158226 10.1152/jn.1994.71.1.17

[B21] De-Miguel F. F. Fuxe K. (2012). Extrasynaptic neurotransmission as a way of modulating neuronal functions. Front. Physiol. 3:16 10.3389/fphys.2012.00016 22363292 PMC3279940

[B22] Dodt H. U. Frick A. Kampe K. Zieglgansberger W. (1998). NMDA and AMPA receptors on neocortical neurons are differentially distributed. Eur. J. Neurosci. 10, 3351–3357 10.1046/j.1460-9568.1998.00338.x 9824448

[B23] Elston G. N. (2003). Cortex, cognition and the cell: new insights into the pyramidal neuron and prefrontal function. Cereb. Cortex 13, 1124–1138 10.1093/cercor/bhg093 14576205

[B24] Enoki R. Kiuchi T. Koizumi A. Sasaki G. Kudo Y. Miyakawa H. (2004). NMDA receptor-mediated depolarizing after-potentials in the basal dendrites of CA1 pyramidal neurons. Neurosci. Res. 48, 325–333 10.1016/j.neures.2003.11.011 15154678

[B25] Gasparini S. Migliore M. Magee J. C. (2004). On the initiation and propagation of dendritic spikes in CA1 pyramidal neurons. J. Neurosci. 24, 11046–11056 10.1523/JNEUROSCI.2520-04.2004 15590921 PMC6730267

[B26] Geisler W. S. Albrecht D. G. (1995). Bayesian analysis of identification performance in monkey visual cortex: nonlinear mechanisms and stimulus certainty. Vision Res. 35, 2723–2730 10.1016/0042-6989(95)00029-Y 7483312

[B27] Harris A. Z. Pettit D. L. (2007). Extrasynaptic and synaptic NMDA receptors form stable and uniform pools in rat hippocampal slices. J. Physiol. (Lond.) 584, 509–519 10.1113/jphysiol.2007.137679 17717018 PMC2277145

[B28] Herman M. A. Nahir B. Jahr C. E. (2011). Distribution of extracellular glutamate in the neuropil of hippocampus. PLoS ONE 6, e26501 10.1371/journal.pone.0026501 22069455 PMC3206024

[B29] Hodgkin A. L. Huxley A. F. (1952). A quantitative description of membrane current and its application to conduction and excitation in nerve. J. Physiol. (Lond.) 117, 500–544 12991237 10.1113/jphysiol.1952.sp004764 PMC1392413

[B30] Holthoff K. Kovalchuk Y. Yuste R. Konnerth A. (2004). Single-shock LTD by local dendritic spikes in pyramidal neurons of mouse visual cortex. J. Physiol. (Lond.) 560, 27–36 10.1113/jphysiol.2004.072678 15319420 PMC1665193

[B31] Jia H. Rochefort N. L. Chen X. Konnerth A. (2010). Dendritic organization of sensory input to cortical neurons in vivo. Nature 464, 1307–1312 10.1038/nature08947 20428163

[B32] Jourdain P. Bergersen L. H. Bhaukaurally K. Bezzi P. Santello M. Domercq M. Matute C. Tonello F. Gundersen V. Volterra A. (2007). Glutamate exocytosis from astrocytes controls synaptic strength. Nat. Neurosci. 10, 331–339 10.1038/nn1849 17310248

[B33] Kampa B. M. Stuart G. J. (2006). Calcium spikes in basal dendrites of layer 5 pyramidal neurons during action potential bursts. J. Neurosci. 26, 7424–7432 10.1523/JNEUROSCI.3062-05.2006 16837590 PMC6674200

[B34] Katona G. Kaszas A. Turi G. F. Hajos N. Tamas G. Vizi E. S. Rozsa B. (2011). Roller Coaster Scanning reveals spontaneous triggering of dendritic spikes in CA1 interneurons. Proc. Natl. Acad. Sci. U.S.A. 108, 2148–2153 10.1073/pnas.1009270108 21224413 PMC3033272

[B35] Kleindienst T. Winnubst J. Roth-Alpermann C. Bonhoeffer T. Lohmann C. (2011). Activity-dependent clustering of functional synaptic inputs on developing hippocampal dendrites. Neuron 72, 1012–1024 10.1016/j.neuron.2011.10.015 22196336

[B36] Korngreen A. Sakmann B. (2000). Voltage-gated K+ channels in layer 5 neocortical pyramidal neurones from young rats: subtypes and gradients. J. Physiol. 3, 621–639 10.1111/j.1469-7793.2000.00621.x 10856117 PMC2269970

[B37] Kullmann D. M. Min M. Y. Asztely F. Rusakov D. A. (1999). Extracellular glutamate diffusion determines the occupancy of glutamate receptors at CA1 synapses in the hippocampus. Philos. Trans. R. Soc. Lond. B Biol. Sci. 354, 395–402 10.1098/rstb.1999.0392 10212489 PMC1692494

[B38] Kurashige H. Cateau H. (2011). Dendritic slow dynamics enables localized cortical activity to switch between mobile and immobile modes with noisy background input. PLoS ONE 6, e24007 10.1371/journal.pone.0024007 21931635 PMC3169558

[B39] Larkman A. U. (1991). Dendritic morphology of pyramidal neurones of the visual cortex of the rat: III. Spine distributions. J. Comp. Neurol. 306, 332–343 10.1002/cne.903060208 1711059

[B40] Larkum M. E. Nevian T. (2008). Synaptic clustering by dendritic signalling mechanisms. Curr. Opin. Neurobiol. 18, 321–331 10.1016/j.conb.2008.08.013 18804167

[B41] Larkum M. E. Nevian T. Sandler M. Polsky A. Schiller J. (2009). Synaptic integration in tuft dendrites of layer 5 pyramidal neurons: a new unifying principle. Science 325, 756–760 10.1126/science.1171958 19661433

[B42] Lewis B. L. O’Donnell P. (2000). Ventral tegmental area afferents to the prefrontal cortex maintain membrane potential ‘up’ states in pyramidal neurons via D(1) dopamine receptors. Cereb. Cortex 10, 1168–1175 10.1093/cercor/10.9.873 11073866

[B43] London M. Segev I. (2001). Synaptic scaling in vitro and in vivo. Nat. Neurosci. 4, 853–855 10.1038/nn0901-853 11528406

[B44] Losonczy A. Makara J. K. Magee J. C. (2008). Compartmentalized dendritic plasticity and input feature storage in neurons. Nature 452, 436–441 10.1038/nature06725 18368112

[B45] Magee J. C. (2011). Observations on clustered synaptic plasticity and highly structured input patterns. Neuron 72, 887–888 10.1016/j.neuron.2011.12.009 22196323

[B46] Major G. Polsky A. Denk W. Schiller J. Tank D. W. (2008). Spatiotemporally graded NMDA spike/plateau potentials in basal dendrites of neocortical pyramidal neurons. J. Neurophysiol. 99, 2584–2601 10.1152/jn.00011.2008 18337370

[B47] Mayer M. L. Westbrook G. L. Guthrie P. B. (1984). Voltage-dependent block by Mg2+ of NMDA responses in spinal cord neurones. Nature 309, 261–263 10.1038/309261a0 6325946

[B48] Milojkovic B. A. Radojicic M. S. Antic S. D. (2005a). A strict correlation between dendritic and somatic plateau depolarizations in the rat prefrontal cortex pyramidal neurons. J. Neurosci. 25, 3940–3951 10.1523/JNEUROSCI.5314-04.2005 15829646 PMC5643048

[B49] Milojkovic B. A. Wuskell J. P. Loew L. M. Antic S. D. (2005b). Initiation of sodium spikelets in basal dendrites of neocortical pyramidal neurons. J. Membr. Biol. 208, 155–169 10.1007/s00232-005-0827-7 16645744 PMC5652330

[B50] Milojkovic B. A. Radojicic M. S. Goldman-Rakic P. S. Antic S. D. (2004). Burst generation in rat pyramidal neurones by regenerative potentials elicited in a restricted part of the basilar dendritic tree. J. Physiol. (Lond.) 558, 193–211 10.1113/jphysiol.2004.061416 15155788 PMC1664906

[B51] Milojkovic B. A. Zhou W. L. Antic S. D. (2007). Voltage and calcium transients in basal dendrites of the rat prefrontal cortex. J. Physiol. (Lond.) 585, 447–468 10.1113/jphysiol.2007.142315 17932150 PMC2375496

[B52] Min R. Nevian T. (2012). Astrocyte signaling controls spike timing-dependent depression at neocortical synapses. Nat. Neurosci. 15, 746–753 10.1038/nn.3075 22446881

[B53] Moore A. R. Zhou W. L. Potapenko E. S. Kim E. J. Antic S. D. (2011). Brief dopaminergic stimulations produce transient physiological changes in prefrontal pyramidal neurons. Brain Res. 1370, 1–15 10.1016/j.brainres.2010.10.111 21059342 PMC3019254

[B54] Morita K. (2008). Possible role of dendritic compartmentalization in the spatial working memory circuit. J. Neurosci. 28, 7699–7724 10.1523/JNEUROSCI.3948-07.2008 18650346 PMC6670839

[B55] Nimchinsky E. A. Yasuda R. Oertner T. G. Svoboda K. (2004). The number of glutamate receptors opened by synaptic stimulation in single hippocampal spines. J. Neurosci. 24, 2054–2064 10.1523/JNEUROSCI.5066-03.2004 14985448 PMC6730404

[B56] Nizami L. (2005). Dynamic range relations for auditory primary afferents. Hear. Res. 208, 26–46 10.1016/j.heares.2005.05.002 16005586

[B57] Nowak L. Bregestovski P. Ascher P. Herbet A. Prochiantz A. (1984). Magnesium gates glutamate-activated channels in mouse central neurones. Nature 307, 462–465 10.1038/307462a0 6320006

[B58] Oakley J. C. Schwindt P. C. Crill W. E. (2001). Dendritic calcium spikes in layer 5 pyramidal neurons amplify and limit transmission of ligand-gated dendritic current to soma. J. Neurophysiol. 86, 514–527 11431529 10.1152/jn.2001.86.1.514

[B59] Parpura V. Basarsky T. A. Liu F. Jeftinija K. Jeftinija S. Haydon P. G. (1994). Glutamate-mediated astrocyte-neuron signalling. Nature 369, 744–747 10.1038/369744a0 7911978

[B60] Persi E. Hansel D. Nowak L. Barone P. van Vreeswijk C. (2011). Power-law input-output transfer functions explain the contrast-response and tuning properties of neurons in visual cortex. PLoS Comput. Biol. 7, e1001078 10.1371/journal.pcbi.1001078 21390280 PMC3044767

[B61] Poirazi P. Brannon T. Mel B. W. (2003). Pyramidal neuron as two-layer neural network. Neuron 37, 989–999 10.1016/S0896-6273(03)00149-1 12670427

[B62] Polsky A. Mel B. Schiller J. (2009). Encoding and decoding bursts by NMDA spikes in basal dendrites of layer 5 pyramidal neurons. J. Neurosci. 29, 11891–11903 10.1523/JNEUROSCI.5250-08.2009 19776275 PMC3850222

[B63] Polsky A. Mel B. W. Schiller J. (2004). Computational subunits in thin dendrites of pyramidal cells. Nat. Neurosci. 7, 621–627 10.1038/nn1253 15156147

[B64] Remy S. Csicsvari J. Beck H. (2009). Activity-dependent control of neuronal output by local and global dendritic spike attenuation. Neuron 61, 906–916 10.1016/j.neuron.2009.01.032 19323999

[B65] Rhodes P. (2006). The properties and implications of NMDA spikes in neocortical pyramidal cells. J. Neurosci. 26, 6704–6715 10.1523/JNEUROSCI.3791-05.2006 16793878 PMC6673826

[B66] Schaefer A. T. Helmstaedter M. Schmitt A. C. Bar-Yehuda D. Almog M. Ben-Porat H. Sakmann B. Korngreen A. (2007). Dendritic voltage-gated K+ conductance gradient in pyramidal neurones of neocortical layer 5B from rats. J. Physiol. (Lond.) 579, 737–752 10.1113/jphysiol.2006.122564 17158172 PMC2151356

[B67] Schiller J. Helmchen F. Sakmann B. (1995). Spatial profile of dendritic calcium transients evoked by action potentials in rat neocortical pyramidal neurones. J. Physiol. 487, 583–600 8544123 10.1113/jphysiol.1995.sp020902 PMC1156647

[B68] Schiller J. Major G. Koester H. J. Schiller Y. (2000). NMDA spikes in basal dendrites of cortical pyramidal neurons. Nature 404, 285–289 10.1038/35005094 10749211

[B69] Suzuki T. Kodama S. Hoshino C. Izumi T. Miyakawa H. (2008). A plateau potential mediated by the activation of extrasynaptic NMDA receptors in rat hippocampal CA1 pyramidal neurons. Eur. J. Neurosci. 28, 521–534 10.1111/j.1460-9568.2008.06324.x 18702724

[B70] Swadlow H. A. Hicks T. P. (1997). Subthreshold receptive fields and baseline excitability of “silent” S1 callosal neurons in awake rabbits: contributions of AMPA/kainate and NMDA receptors. Exp. Brain Res. 115, 403–409 10.1007/PL00005710 9262195

[B71] Timofeev I. Grenier F. Steriade M. (2001). Disfacilitation and active inhibition in the neocortex during the natural sleep-wake cycle: an intracellular study. Proc. Natl. Acad. Sci. U.S.A. 98, 1924–1929 10.1073/pnas.98.4.1924 11172052 PMC29358

[B72] Tovar K. R. Westbrook G. L. (1999). The incorporation of NMDA receptors with a distinct subunit composition at nascent hippocampal synapses in vitro. J. Neurosci. 19, 4180–4188 10234045 10.1523/JNEUROSCI.19-10-04180.1999 PMC6782704

[B73] Varga Z. Jia H. Sakmann B. Konnerth A. (2011). Dendritic coding of multiple sensory inputs in single cortical neurons in vivo. Proc. Natl. Acad. Sci. U.S.A. 108, 15420–15425 10.1073/pnas.1112355108 21876170 PMC3174623

[B74] Waters J. Helmchen F. (2004). Boosting of action potential backpropagation by neocortical network activity in vivo. J. Neurosci. 24, 11127–11136 10.1523/JNEUROSCI.2933-04.2004 15590929 PMC6730284

[B75] Wei D. S. Mei Y. A. Bagal A. Kao J. P. Thompson S. M. Tang C. M. (2001). Compartmentalized and binary behavior of terminal dendrites in hippocampal pyramidal neurons. Science 293, 2272–2275 10.1126/science.1061198 11567143

[B76] Wilson C. J. Kawaguchi Y. (1996). The origins of two-state spontaneous membrane potential fluctuations of neostriatal spiny neurons. J. Neurosci. 16, 2397–2410 8601819 10.1523/JNEUROSCI.16-07-02397.1996 PMC6578540

[B77] Yasuda R. Sabatini B. L. Svoboda K. (2003). Plasticity of calcium channels in dendritic spines. Nat. Neurosci. 6, 948–955 10.1038/nn1112 12937422

[B78] Zhou W. L. Yan P. Wuskell J. P. Loew L. M. Antic S. D. (2008). Dynamics of action potential backpropagation in basal dendrites of prefrontal cortical pyramidal neurons. Eur. J. Neurosci. 27, 1–14 10.1111/j.1460-9568.2008.06075.x 18279369 PMC2715167

